# Consumption of flavonoids and risk of hormone-related cancers: a systematic review and meta-analysis of observational studies

**DOI:** 10.1186/s12937-022-00778-w

**Published:** 2022-05-11

**Authors:** Fubin Liu, Yu Peng, Yating Qiao, Yubei Huang, Fengju Song, Ming Zhang, Fangfang Song

**Affiliations:** 1 Department of Epidemiology and Biostatistics, Key Laboratory of Molecular Cancer Epidemiology, Tianjin Medical University Cancer Institute and Hospital, Tianjin, 300060 China; 2 Key Laboratory of Cancer Prevention and Therapy, Tianjin Medical University Cancer Institute and Hospital, Tianjin, 300060 China; 3 Key Laboratory of Breast Cancer Prevention and Therapy, Ministry of Education, National Clinical Research Center for Cancer, Tianjin’s Clinical Research Center for Cancer, Tianjin Medical University Cancer Institute and Hospital, Tianjin, 300060 China; 4 Shenzhen Prevention and Treatment Center for Occupational Diseases, Shenzhen, 518020 Guangdong China

**Keywords:** Flavonoids, Flavonoid subclasses, Hormone-related cancers, Observational studies, Meta-analysis

## Abstract

**Background:**

Flavonoids seem to have hormone-like and anti-hormone properties so that the consumption of flavonoids may have potential effects on hormone-related cancers (HRCs), but the findings have been inconsistent so far. This meta-analysis was aimed to explore the association between flavonoids intake and HRCs risk among observational studies.

**Methods:**

Qualified articles, published on PubMed, EMBASE, and China National Knowledge Infrastructure (CNKI) from January 1999 to March 2022 and focused on relationships between flavonoids (total, subclass of and individual flavonoids) and HRCs (breast, ovarian, endometrial, thyroid, prostate and testicular cancer), were retrieved for pooled analysis. Random effects models were performed to calculate the pooled odds ratios (ORs) and corresponding 95% confidence intervals (CIs). Funnel plots and Begg’s/Egger’s test were used to evaluate the publication bias. Subgroup analyses and sensitivity analyses were conducted to explore the origins of heterogeneity.

**Results:**

All included studies were rated as medium or high quality. Higher consumption of flavonols (OR = 0.85, 95% CI: 0.76–0.94), flavones (OR = 0.85, 95% CI: 0.77–0.95) and isoflavones (OR = 0.87, 95% CI: 0.82–0.92) was associated with a decreased risk of women-specific cancers (breast, ovarian and endometrial cancer), while the higher intake of total flavonoids was linked to a significantly elevated risk of prostate cancer (OR = 1.11, 95% CI: 1.02–1.21). A little evidence implied that thyroid cancer risk was augmented with the higher intake of flavones (OR = 1.24, 95% CI: 1.03–1.50) and flavanones (OR = 1.31, 95% CI: 1.09–1.57).

**Conclusions:**

The present study suggests evidence that intake of total flavonoids, flavonols, flavones, flavanones, flavan-3-ols and isoflavones would be associated with a lower or higher risk of HRCs, which perhaps provides guidance for diet guidelines to a certain extent.

**Trial registration:**

This protocol has been registered on PROSPERO with registration number CRD42020200720.

**Supplementary Information:**

The online version contains supplementary material available at 10.1186/s12937-022-00778-w.

## Introduction

Hormone-related cancers (HRCs) are greatly influenced by hormone levels and generally respond to hormone regulation, which plays an indispensable role in tumor growth. Six cancer types, including breast, ovarian, endometrial, thyroid, prostate and testicular cancer, are usually referred to as HRCs since they share the same carcinogenic mechanism [1]. Worldwide, there were about 4.7 million newly diagnosed cases of HRCs in 2018, accounting for more than a quarter of new cancer cases. As the most frequently diagnosed cancer in the great majority of countries, HRCs have become the leading cause of cancer death in over 100 countries [2]. With a cumulative knowledge for the role of hormones in HRCs, hormonal therapy is increasingly important [3, 4], but remains controversial due to more or less inevitable side-effects in the treatment course [5, 6]. Encouragingly, diets rich in vegetables, fruits and tea are found to reduce the cancer risk [7], having the potential to exert chemopreventive effects with the presence of anticarcinogenic phytochemicals [8].

Flavonoids represent one of the largest groups of phytochemicals, as a class of polyphenols with a basic benzo-γ-pyrone structure widely distributed in the plant kingdom, which mainly exist in leaves, flowers, roots, stems, and fruits of plants [9]. The U.S. Department of Agriculture (USDA) database categorizes flavonoids into six subclasses: flavonols, flavones, flavanones, anthocyanidins, flavan-3-ols and isoflavones [10, 11]. Allegedly, flavonoids have multiple functions in different physiological and pathological process of cancer, such as tumor cell proliferation, inflammation, angiogenesis, invasion and metastasis [12–14]. Besides, flavonoids seem to have hormone-like and anti-hormone effects due to their structural similarity to the natural estrogen estradiol, as well as other steroid hormones and steroid hormone antagonists [15]. Further, there is evidence that certain flavonoids may inhibit the activity of aromatase, an enzyme that catalyzes the conversion of androgen to estrogen, and up-regulation of the latter can promote the development of HRCs [16].

Most of these studies on flavonoids and HRCs have been done in vivo or in vitro, but less in humans. The evidence for the relationship between intake of flavonoids and risk of the aforementioned HRCs (except testicular cancer) among observational studies has been summed up by several existing meta-analyses [17–24]. It is noteworthy that these meta-analyses were either only based on individual HRCs type, or just focused on specific flavonoid subclass such as isoflavones, and lack of consistency because of the different methods to assess flavonoids intake. The criteria for evaluating flavonoids intake were not stringent enough, leading to the inclusion of low-quality studies.

As of the last summary of evidence, new research has come out, and an outline of the effects of total, subclass of, and individual flavonoids intake on HRCs has so far not been available. Thus, in this study, a meta-analysis was carried out for a comprehensive evaluation of total/subclass of/individual flavonoids to summarize epidemiological findings and to elucidate the association between flavonoids intake and HRCs risk.

## Methods

### Search strategy

A systematic search on PubMed (http://www.ncbi.nlm.nih.gov/pubmed/) and EMBASE (http://www.embase.com/) databases for English studies and on China National Knowledge Infrastructure (CNKI, https://www.cnki.net/) for Chinese articles published through January 1999 up to March 2022 was performed. The search terms used for retrieval were as follows: ((“flavonoids”[MeSH Terms] OR “flavonoid$”[All Fields]) OR (“flavanones”[MeSH Terms] OR “flavanone$”[All Fields]) OR (“isoflavones”[MeSH Terms] OR “isoflavone$”[All Fields] OR “isoflavonoid$”[all fields]) OR (“flavones”[MeSH Terms] OR “flavone$”[All Fields]) OR (“flavan-3-ol$”[All Fields] OR “flavanol$”[All Fields]) OR (“flavonols”[MeSH Terms] OR “flavonol$”[All Fields]) OR “anthocyanidins”[all fields]) AND (“breast”[all fields] OR (“ovarian”[all fields] OR “ovary”[all fields]) OR “endometrial”[all fields] OR “prostate”[all fields] OR (“testis”[all fields] OR “testicular”[all fields] OR “testicle”[all fields]) OR “thyroid”[all fields]) AND (“neoplasms”[MeSH Terms] OR “neoplasms”[All Fields] OR “cancer”[All Fields]) AND (“Epidemiologic Studies”[MeSH Terms]) AND (“1999/1/1”[Date—Publication]: “2022/3/31”[Date—Publication]). Simultaneously, we inspected references cited in retrieved articles to recognize extra studies and undertook to get access to unpublished data. The meta-analysis was conducted following the Preferred Reporting Items for Systematic Reviews and Meta-Analyses (PRISMA) guidelines. This protocol has been registered on PROSPERO with registration number CRD42020200720 (https://www.crd.york.ac.uk/prospero/).

### Inclusion and exclusion criteria

Eligible studies for the pooled analysis were selected as the following criteria: (i) an observational study (an prospective design such as cohort study or nested case–control study, or retrospective design namely case–control study); (ii) the association between intake of total/subclass of/individual flavonoids and the risk of any HRCs was evaluated; (iii) relative risks (RRs), odds ratios (ORs) or hazard ratios (HRs) with the corresponding 95% confidence intervals (CIs) for the highest category of exposure were reported; (iv) evaluating flavonoids consumption by dietary questionnaires and measurement for serum, plasma and urine. Only the research with the longest follow-up period was taken into consideration when multiple articles involving the same cohort have been produced.

The exclusion criteria were as described below: (i) out of adequate statistics; (ii) incomplete evaluation on flavonoids (no total and subclass of flavonoids or less than 3 kinds of flavonoid); (iii) unconfirmed comparison and comparison by per each increase for flavonoids intake; (iv) reported as an abstract, summary, comments, review or editorial; (v) flavonoids intake from sources other than dietary questionnaires, serum, plasma and urine; (vi) amount of soy protein or soy foods or soy supplements consumed as a representative of dietary isoflavone intake. The selection process and the examination for retrieved articles were performed independently by two authors (F. Liu and Y. Peng). Inconsistencies were referred to another author (Yating Qiao) for resolution.

### Data extraction

Two authors (F. Liu and Y. Peng) reviewed full-text articles separately, and extracted the following characteristics from the original: first author’s name, publication year, study design (cohort, case–control or nested case–control study), study region (country or area), sex and age range, number of cases and controls or total cohort participants with follow-up period, exposure measurement, the most adjusted ORs in case–control studies or HRs/RRs in cohort studies and the corresponding 95% CIs comparing the highest vs. lowest (or reference) level of flavonoids intake, and the covariates in multivariable models. Dissents on data extraction were settled through argumentum.

### Quality assessment

The Newcastle–Ottawa Scale (NOS) quality assessment criteria, consisting of a total of 9 points in three dimensions (4 points in “selection”, 2 points in “comparability” and 3 points in “outcome” for cohort studies or “exposure” for case–control studies), was employed to appraise the quality of selected study [25]. Studies assigned 7–9, 4–6, and 0–3 points were recognized as high-, medium-, and low-quality, respectively (higher score representing higher quality).

### Statistical analysis

Statistical analyses were performed by using Stata 15.1 software (StataCorp LP, College Station, TX). ORs with 95% CIs were assessed for ascertaining the association between flavonoids intake and HRCs risk. The merged ORs with 95% CIs calculated by the most adjusted HRs, RRs or ORs with 95% CIs announced in the original were illustrated in forest plots using random effects models depicted by DerSimonian-Laird method [26]. Statistic *I*^*2*^ was applied for the evaluation of heterogeneity among studies [27] and the two-sided *p* values based on the *Q* test of heterogeneity were reported (*p* < 0.05 suggested significant heterogeneity). Publication biases were evaluated by funnel plots, and calculated by Begg’s tests [28] and Egger’s tests [29]. Besides, to explore the source of heterogeneity among the studies, sensitivity analyses were preferred to evaluate the robustness of the results of the combined effects, which were achieved by sequential removal of each study. Further, subgroup analyses were implemented based on study design, menopausal status (in breast cancer) and study region.

## Results

### Study characteristics

**Fig. 1:**
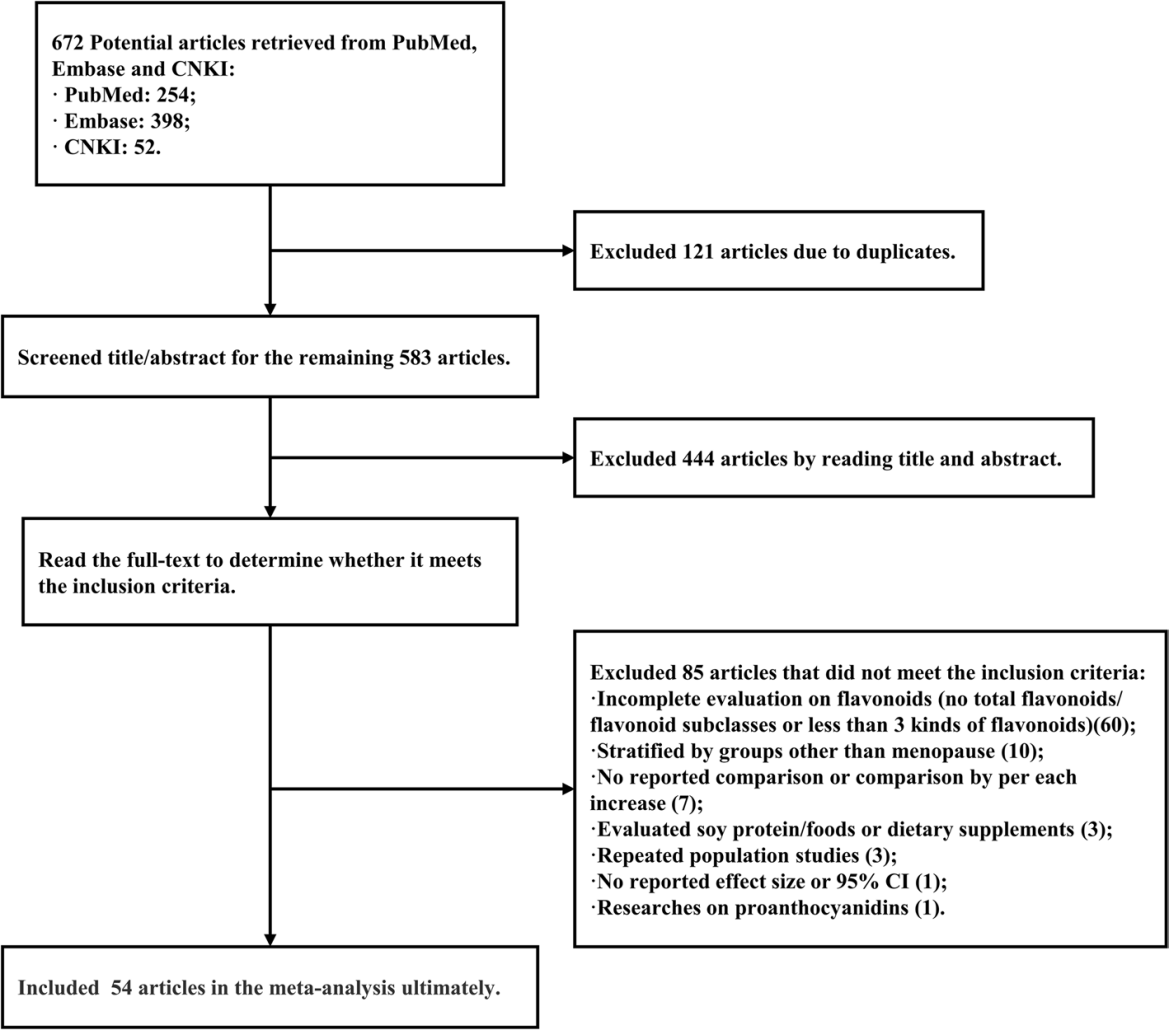
Flowchart for retrieving articles and selecting eligible studies. *CNKI, China National Knowledge Infrastructure; CI, confidence interval

A flowchart of the literature screening process was outlined in Fig. 1, and the details of the selected eligible studies were listed in Supplementary Table S1. Fifty-four published articles contained 51 studies. It consisted of 22 prospective cohort studies, 1 nested case–control study, 18 population-based case–control studies and 10 hospital-based case–control studies. Urine samples were collected in three studies and serum samples were collected in two. The remaining studies assessed flavonoid intake using dietary questionnaires. Most of these studies made adjustment for multiple latent confounding factors, including demographics like age, body mass index (BMI), smoking status, alcohol intake, family history of cancer, energy/caloric intake, as well as female reproduction details such as age at menarche/first birth/menopause and parity, which were confined to women-specific cancers. On assessment of the methodological quality using the NOS, all studies attained the score 5 or above, namely all included studies were of medium- or high-quality (Supplementary Tables S2 and S3).

### Flavonoids intake and risk of hormone-related cancers

#### Female breast cancer

**Fig. 2:**
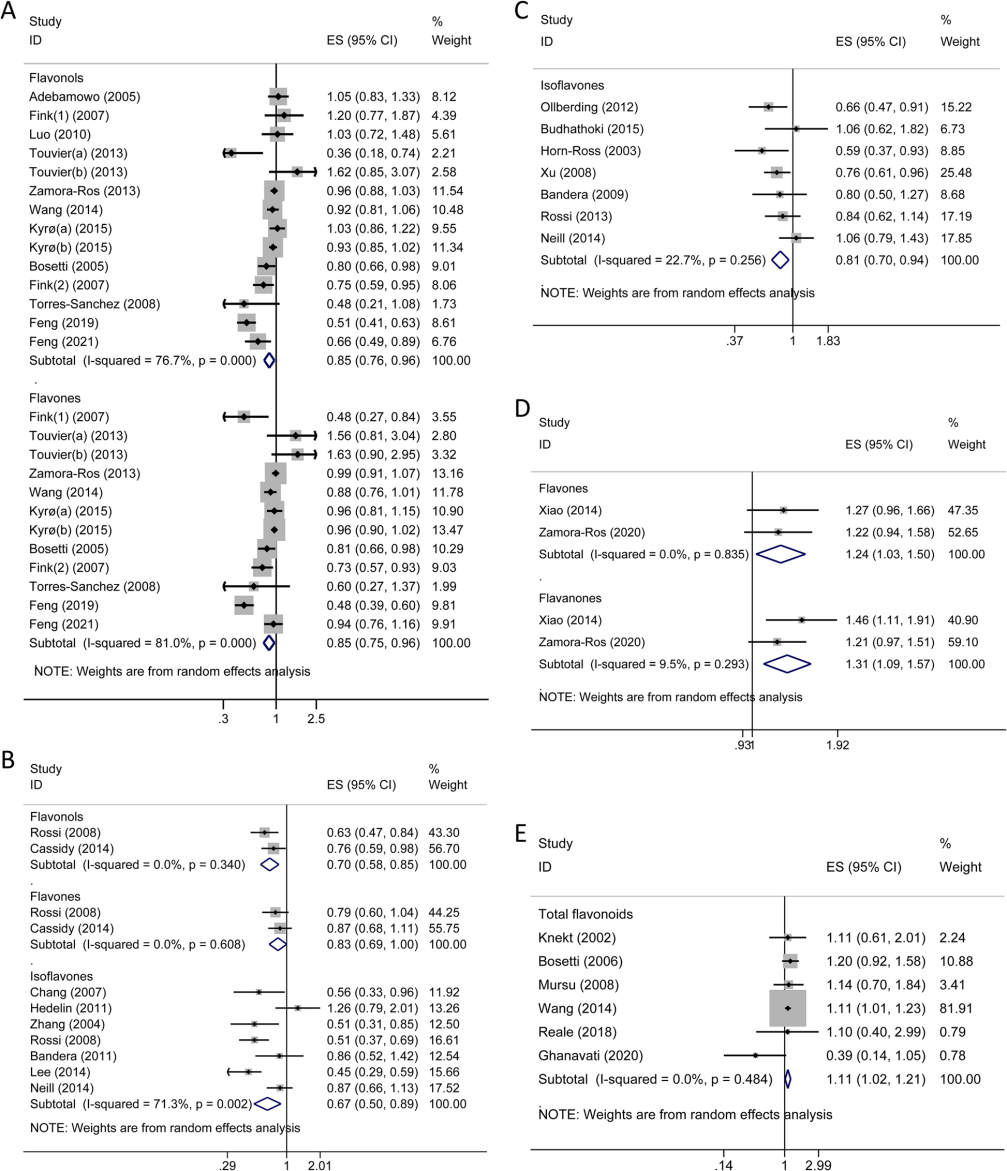
Forest plots for the association between total/subclass of flavonoids consumption and hormone-related cancer risk. **A** breast cancer; **B** ovarian cancer; **C** endometrial cancer; **D** thyroid cancer; **E** prostate cancer. Use (1) and (2) to distinguish two different studies by the same author in the same year; two datasets of the same study were represented by (a) and (b). *ES, effect size; CI, confidence interval

**Fig. 3:**
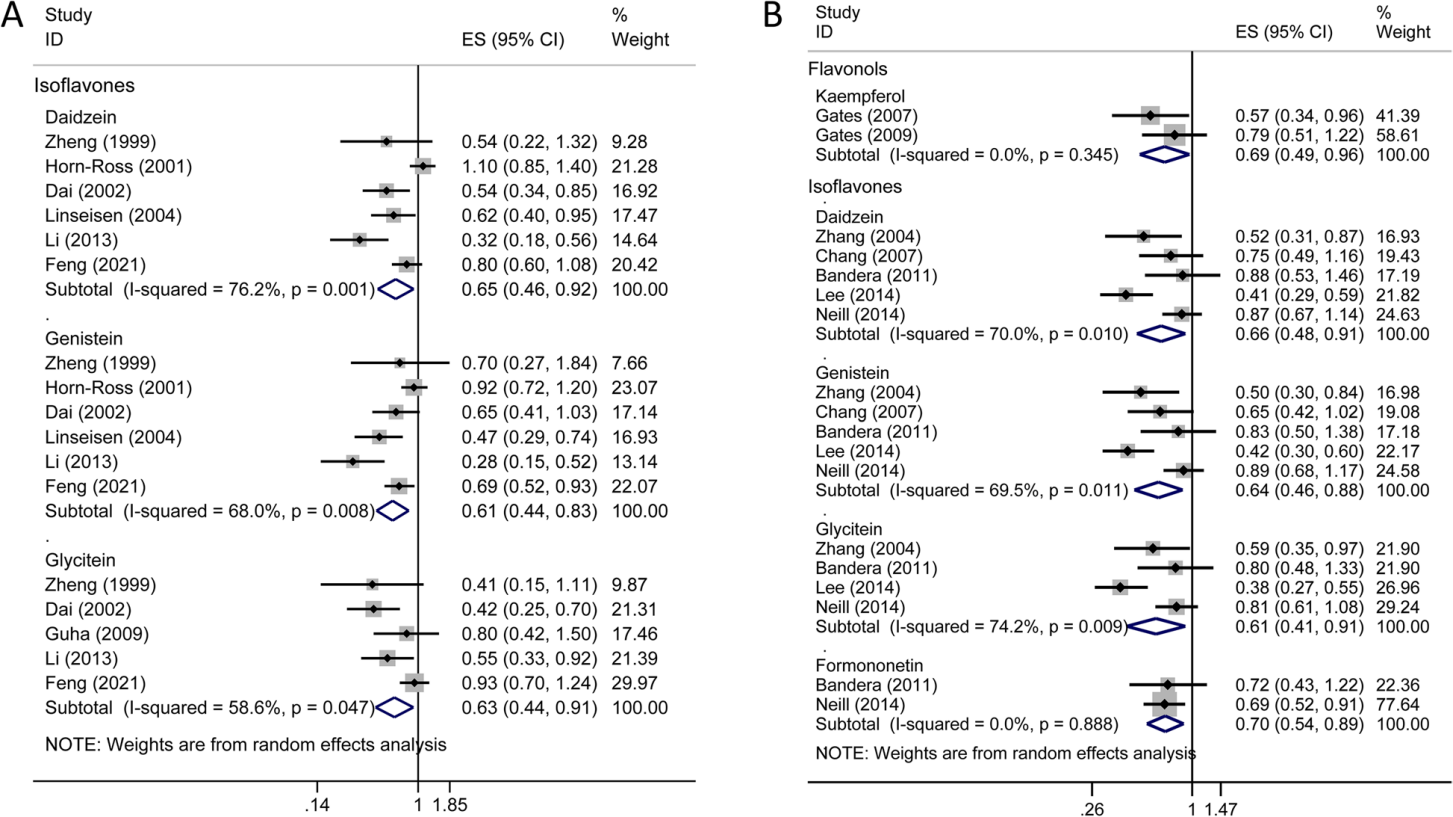
Forest plots for the association between individual flavonoid compounds consumption and hormone-related cancer risk. **A** breast cancer; **B** ovarian cancer. *ES, effect size; CI, confidence interval

Nineteen studies (9 cohort studies, 10 case–control studies and 1 nested case–control studies) [30–50] were conducted on breast cancer (Supplementary Table S4). Overall, no significant effect of total flavonoids intake on breast cancer risk was observed. The risk of breast cancer significantly decreased in women with higher consumption of flavonols (OR = 0.85; 95% CI, 0.76–0.96; *I*^*2*^ = 76.7%; *p* < 0.001) and flavones (OR = 0.85; 95% CI, 0.75–0.96; *I*^*2*^ = 81.0%; *p* < 0.001), compared with that in those with lower consumption (Fig. 2A). These protective effects seemed more pronounced in case–control studies but not in prospective studies. In subgroup analyses, the significant association of flavones with reduced risk of breast cancer was observed in premenopausal women but not in postmenopausal women; Flavonols were the opposite (Supplementary Table S5). Moreover, based on study region, higher consumption of flavones was linked to a decreased risk of breast cancer in non-Asians whereas flavanones and isoflavones had a protective effect only in Asians (Supplementary Table S6). Null results were found for individual compounds of flavonols, flavanones and flavan-3-ols while significant decreased summary risk estimates were retrieved from main individual isoflavones (daidzein, OR = 0.65, 95% CI 0.46–0.92; genistein, OR = 0.61, 95% CI 0.44–0.83; and glycitein, OR = 0.63, 95% CI 0.44–0.91; Fig. 3A and Supplementary Table S4).

#### Ovarian cancer

As shown in Fig. 2B and Supplementary Table S4, the combined analyses of the five cohort studies and six case–control studies [7, 40, 51–59] indicated that ovarian cancer risk was significantly reduced in women with the higher intake of flavonols (OR = 0.70; 95% CI, 0.58–0.85; *I*^*2*^ = 0.0%; *p* = 0.340), and isoflavones (OR = 0.67; 95% CI, 0.50–0.89; *I*^*2*^ = 71.3%; *p* = 0.002) respectively, but not with higher consumption of total flavonoids and other flavonoid subclasses. And, subgroup analysis suggested isoflavones might be more phylactic against ovarian cancer in case–control studies and in Asian populations (Supplementary Table S6). For individual flavonoids, intake of both kaempferol from the flavonol subclass and all the four individual isoflavones (daidzein, genistein, glycitein and formononetin) contributed to a significantly decreased risk of ovarian cancer (Fig. 3B and Supplementary Table S4).

#### Endometrial cancer

Three prospective and 5 case–control studies [40, 51, 60–65] of total flavonoids and/or isoflavones subclass were conducted on endometrial cancer. No significant association with endometrial cancer risk was observed for total flavonoids (OR = 0.93; 95% CI, 0.67–1.27; *I*^*2*^ = 32.7%; *p* = 0.223). Whereas, there was a significant association of isoflavone consumption with reduced risk of endometrial cancer (OR = 0.81; 95% CI, 0.70–0.94; *I*^*2*^ = 22.7%; *p* = 0.256; Fig. 2C and Supplementary Table S4), which was limited to case–control studies and non-Asian studies by subgroup analyses (Supplementary Table S6). Further, no significant association between individual isoflavone intake and endometrial cancer risk was observed.

#### Thyroid cancer

Although limited epidemiological studies (2 prospective and 1 case–control studies) [66–68] have been launched to evaluate the flavonoids intake on thyroid cancer risk, we still observed an increment in thyroid cancer risk conferred by the higher intake of flavones (OR = 1.24; 95% CI, 1.03–1.50; *I*^*2*^ = 0%; *p* = 0.835) and flavanones (OR = 1.31; 95% CI, 1.09–1.57; *I*^*2*^ = 9.5%; *p* = 0.293) with no evidence of heterogeneity, respectively (Fig. 2D and Supplementary Table S4).

#### Prostate cancer

Figure 2E and Supplementary Table S4 demonstrated the summarized risk estimates for six cohort and six case–control studies [31, 69–81] on consumption of flavonoids and prostate cancer risk. We observed an increased risk of prostate cancer by higher intake of total flavonoids (OR = 1.11; 95% CI, 1.02–1.21; *I*^*2*^ = 0%; *p* = 0.484), which was more pronounced in cohort studies. And, this positive relationship was found only in non-Asia populations (Supplementary Table S6). Of note, for the anthocyanidins and flavan-3-ols subclass, the higher intake category presented a markedly elevated prostate cancer risk compared to the lower intake category in prospective studies. For individual compounds, no significant association was attained in our analysis.

#### Testicular cancer

Only one case–control study [82] investigated the consumption of total flavonoids and isoflavones subclass and risk of testicular cancer but showed no significant results (data not shown).

#### Women-specific cancers

A total of 16 aforementioned prospective (15 cohort and 1 nested case–control studies) and 20 case–control studies on breast, endometrial and ovarian cancer were selected for the analysis on the flavonoid intake with the risk of women-specific cancers. No evidence on association between total flavonoids intake and women-specific cancer risk was found in our analysis. For flavonoid subclasses, higher consumption of flavonols (OR = 0.85; 95% CI, 0.76–0.94; *I*^*2*^ = 75.5%; *p* < 0.001), flavones (OR = 0.85; 95% CI, 0.77–0.95; *I*^*2*^ = 76.3%; *p* < 0.001) and isoflavones (OR = 0.87; 95% CI, 0.82–0.92; *I*^*2*^ = 73.8%; *p* < 0.001) was associated with a decreased risk of women-specific cancers, especially among the case–control studies (Supplementary Table S4). Similar results were also observed for the three main individual compounds of isoflavones (daidzein, OR = 0.69, 95% CI 0.58–0.83; genistein, OR = 0.67, 95% CI 0.57–0.80; and glycitein, OR = 0.68, 95% CI 0.57–0.83). In subgroup analyses based on region, the lower women-specific cancer risk was more reported for flavonols (OR = 0.90; 95% CI, 0.82–0.98) and flavones (OR = 0.90; 95% CI, 0.84–0.97) in non-Asian studies, but for flavanones (OR = 0.74; 95% CI, 0.60 to 0.91)and isoflavones (OR = 0.64; 95% CI, 0.55–0.75) in Asians (Supplementary Table S6).

#### Men-specific cancers

Out of the 6 cohort and 7 case–control studies, only the higher consumption of total flavonoids (OR = 1.11; 95% CI, 1.02–1.22; *I*^*2*^ = 0%; *p* = 0.537) was associated with significant increased risk of men-specific cancers including prostate and testicular cancer, which was restricted to prospective studies (Supplementary Table S4).

### Publication bias

**Fig. 4:**
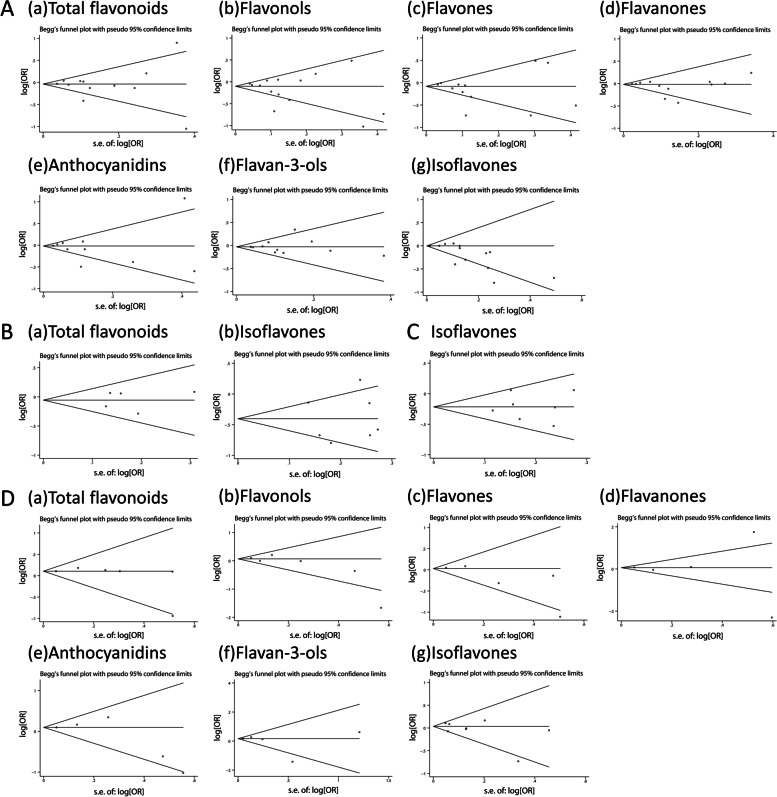
Funnel plots for the association between flavonoid consumption and hormone-related cancer risk. **A** breast cancer; **B** ovarian cancer; **C** endometrial cancer; **D** prostate cancer

As shown in Fig. 4 and Supplementary Table S7, results from both Begg's tests and Egger’s tests indicated little evidence of asymmetry of the funnel plots among total flavonoids and flavonoid subclasses except isoflavones in breast cancer (Begg’s test: *Z* = 2.85, *p*_*Z*_ = 0.004; and Egger’s test: *t* = -2.90, *p*_*t*_ = 0.013) and women-specific cancers (Begg’s test: *Z* = 1.92, *p*_*Z*_ = 0.055; and Egger’s test: *t* = -4.70, *p*_*t*_ < 0.001).

### Sensitivity analysis

**Fig. 5:**
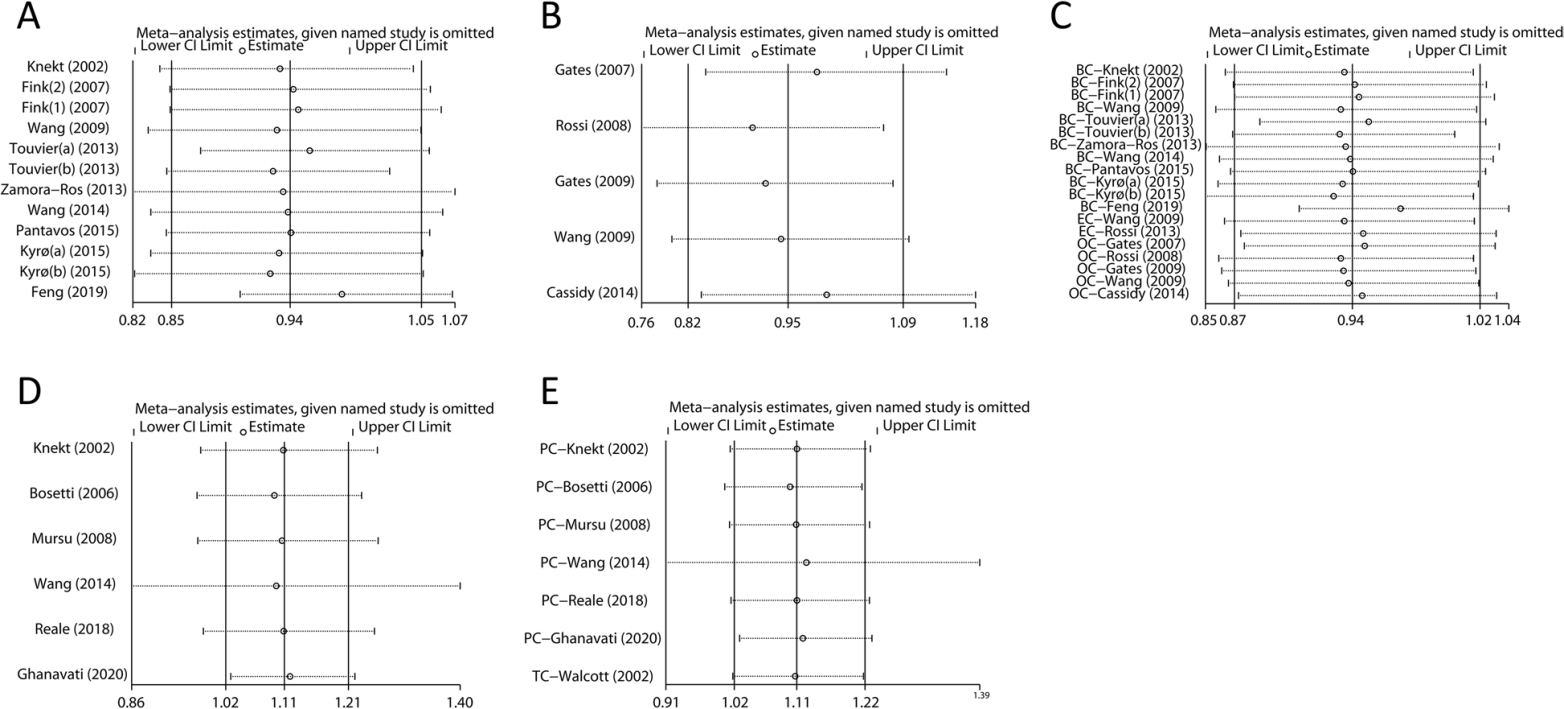
Plots of sensitivity analyses by sequential removal of each study. **A** breast cancer; **B** ovarian cancer; **C** women-specific cancers; **D** prostate cancer; **E** men-specific cancers. *BC, breast cancer; EC, endometrial cancer; OC, ovarian cancer; PC, prostate cancer; TC, testicular cancer

Sensitivity analyses for total flavonoids and HRCs were performed (Fig. 5 and Supplementary Table S8). When any study except Ghanavati’s [81] was eliminated, there was no evidence that total flavonoids had a detrimental effect on prostate cancer. When Wang’ study [77] was eliminated, the dangerous effect of total flavonoids on men-specific cancers disappeared. For other cancers, no statistically significant change in combined effect sizes was detected after excluding each study separately.

## Discussion

In the present study, a comprehensive meta-analysis of existing observational studies on the consumption of flavonoids and risk of HRCs were conducted. The current results showed that higher consumption of total flavonoids was only associated with an increased risk of men-specific cancers, mainly prostate cancer. For the subclasses, flavonols, flavones, and isoflavones, as well as the three main individual compounds of isoflavones (daidzein, genistein and glycitein) may have protective effects on women-specific cancers, whereas potentially dangerous effects of flavones and flavanones have been found in thyroid cancer. There was no evidence in support of any role for anthocyanidins in HRCs.

Flavonoids can interfere with the initiation, promotion and development of cancer through directly inhibiting the process of oxidative stress and oxidative damage [14] and indirectly affecting hormone activity [83]. Aromatase, a rate-limiting enzyme responsible for the biotransformation of estrogen in vivo, can catalyze the transition from androgen to estrogen. Thus, the regulation of estrogen synthesis in vivo by inhibiting aromatase activity has become a way to treat HRCs. Flavanones, flavones and isoflavones, considered to be strong antioxidants due to the phenyl hydroxyl groups on the nucleus of flavonoids, have long been supposed to have the capacity to inhibit the activity of aromatase [84–86]. Some phytoestrogens, such as genistein, apigenin, naringenin, and kaempferol, can bind to estrogen receptors (ER) and compete fiercely with 17β-estradiol in binding to ER [87]. This may be due to the similarity of the flavonoid structure to the polyphenol ring in the steroidal core of 17β-estradiol. Scherbakov et al. found that high dose of phytoestrogens apigenin, genistein, quercetin and naringenin had inhibitory effect on ER ( +) or ER (-) breast cancer cells [88]. Daidzein, genistein, quercetin and luteolin possess the ability of inhibiting the proliferative activity induced by environmental estrogen, which suggests anti-estrogen and anti-cancer functions by such individual compounds of flavonoids [89].

Consistent with the findings herein, Chang et al. [18] implies significant association of flavonols and flavones intake with a reduced risk of breast cancer and a protective effect of flavan-3-ols only in postmenopausal women. Higher intake of kaempferol from the flavonol subclass and isoflavones including daidzein, genistein, glycitein and formononetin was observed to be linked to a decreased risk of ovarian cancer in this analysis, as reported by the two meta-analyses for ovarian cancer [20, 24]. Hua et al. [20] confused isoflavones and quercetin with total flavonoids when evaluating flavonoids intake, which potentially lead to bias. Given that the approach to evaluate flavonoids intake was different from the current research, there was actually no evidence supporting the effect of total flavonoids against ovarian cancer. No striking discordance was found in all pooled analyses for endometrial cancer and prostate cancer among observational studies including this study, demonstrating higher consumption of isoflavones with a decreased risk of endometrial cancer among case–control studies and the higher intake category of total flavonoids with an elevated risk of prostate cancer confined to prospective studies. Nevertheless, a meta-analysis of randomized controlled trials (RCTs) revealed an effect by higher isoflavone intake for a lower risk of prostate cancer [90]. For thyroid cancer, a cohort study evaluating flavonoid subclasses and a case–control study assessing isoflavones only have been reported by Grosso’s meta-analysis [24]. The current analysis added another prospective cohort study evaluating flavonoid subclasses. For flavonoid subclasses except isoflavones, the results of the aforementioned two cohort studies were pooled and uncovered potentially pernicious effects of flavones and flavanones, the latter of which was consistent with Grosso's findings [24].

Menopausal status as an effect modifier may have an influence on the association between flavonoids intake and women-specific cancer risk. Since most studies reported menopausal status limited to breast cancer, the present study conducted subgroup analyses just for breast cancer. A significant association of higher flavones intake in premenopausal women and of higher flavan-3-ols intake in postmenopausal women with reduced risk of breast cancer was found in this study. Isoflavones intake has been reported to be negatively associated with the risk of breast cancer in postmenopausal women [17, 19], but no association was observed in this study. The content of isoflavones in soy products decreases with the processing of raw soybeans. Second-generation foods derived from soy, such as soy bread and biscuits, have much lower levels of isoflavones (2.4 to 18.1 mg/100 g) compared to raw soy flour (exceeds 100 mg/100 g) [21]. Therefore, significant differences in isoflavone component levels in various soy products may lead to different findings. Such inconsistent findings may be attributed to the fact that the study included not soy foods but pure isoflavones when evaluating consumption of isoflavones, which to a certain extent avoided some heterogeneity. Subgroup analyses based on study region were also performed to explore the heterogeneity among studies in different areas. There were great differences between Asian and non-Asian regions in the association between isoflavones intake and the risk of women-specific cancers as well as the flavan-3-ols intake and prostate cancer risk; especially, the results of region-based subgroup analyses limited to breast cancer in up-to-date meta-analyses were consistent with this study [17, 19, 24]. These suggested the existence of an ethnic disparity necessitated for consideration in evaluating the role of flavonoids intake on cancer risks.

Dietary flavonoids are widely present in plant-based foods. Flavonols, the most abundant flavonoids in foods, are mainly found in onion, red wine, olive oil, berries, and grapefruit. The primary sources of flavones, flavanones, flavan-3-ols and anthocyanidins are vegetables, citrus fruits, green tea and colored berries, respectively. Isoflavones are present in significant quantities in legumes mainly soybeans, green beans, mung beans. The bean of the soy plant has been used in both foods and phytotherapeutic supplements. Evidence from pre-clinical studies supports a role for soy/soy isoflavone in reducing the risk of developing prostate cancer and inhibiting its progression [90]. Therefore, as an eminent clinical intervention agent or chemoprevention agent, flavonoids will have great potential for future. In addition, plant-based food consumption and flavonoids intake vary among populations and ethnicities due to regional differences in dietary habits, especially for isoflavones. Higher intake (over 35 ~ 63 mg/d) of isoflavones in Asians tend to be more than ten times of the higher intake (over 0.03 ~ 4 mg/d) in Westerners, due to the quite low soy consumption in the West. Thus a validated and reliable dietary questionnaire for soy isoflavones taking regional variations into account is often required to assess isoflavone intake [19], when setting dietary standards in the future. Moreover, the findings of the present meta-analysis suggested that an intake of approximately more than 14.5 ~ 60 mg/d of flavonols and over 0.2 ~ 31 mg/d of flavones may have a preventive effect on breast cancer.

Due to the wide varieties of available flavonoids, it is tough to assess the consumption of total flavonoids or each kind of flavonoid. The difficulty of measuring flavonoids intake and the limited epidemiological studies made our research to a certain degree of heterogeneity. Most studies used a food frequency questionnaire (FFQ) or a dietary history questionnaire (DHQ) to evaluate flavonoid consumption, which is likely to lead to measurement bias; while other studies assessed exposure on urine or serum levels, but just reflected the short-term exposure of subjects, in consequence, randomness cannot be ignored. Besides, in addition to studies on morbidity, we also included some cohort studies with follow-up outcomes of recurrence or death to jointly probe the association between flavonoids and HRCs, which is presumably one of the origins of heterogeneity.

The research has some advantages that deserve attention. First, this meta-analysis systematically summarized the effective evidence between flavonoids and HRCs. Second, we objectively launched a comprehensive search for studies from 1999 to 2022. Compared with previous meta-analyses, this study included more cohort studies and case–control studies, which undoubtedly provides more tangible and favorable evidence for guiding flavonoids in the prevention of HRCs. Third, most categories of flavonoids were evaluated, including total flavonoids, flavonoid subclasses, and individual compounds in each subclass. Moreover, inclusion and exclusion criteria established were relatively strict. All individual studies included were rated as medium or high quality, which somewhat avoided the insufficiency and unreliability of the results conferred by low-quality studies.

On the contrary, there are some limitations and challenges to be settled in the research. As only observational studies were included, evidence power is not very strong yet. The sample size of individual studies is small and there may still be other unknown confounding factors affecting the results. This limits the opportunities for extrapolation. Further intervention studies are needed to confirm our conclusion. The number of eligible studies is still limited, especially for certain cancers, thus flavonoid subclasses cannot be synthetically analyzed. Further, the criteria for the included studies allowed no subgroup analyses based on dietary and other sources (urine/serum/plasma) of flavonoids to be performed. For the sake of avoiding unnecessary heterogeneity, RCTs were not included in our meta-analysis. Limited clinical trials have prevented our results from providing more conclusive evidence.

## Conclusions

In conclusion, there is a small amount of evidence that total flavonoids, flavonols, flavones, flavanones, flavan-3-ols and isoflavones may be associated with a lower or higher risk of certain HRCs, which maybe provide guidance for diet guidelines to a certain extent in the future. Nonetheless, due to limited data, further prospective studies and a larger number of subjects are warranted to be verified and provide stronger evidence.

## Supplementary Information

**Additional file1.**

## Data Availability

All data generated or analysed during this study are included in this published article [and its supplementary information files].

## Abbreviations

BMI: Body mass index
Cis: Confidence intervals
CNKI: China National Knowledge Infrastructure
DHQ: Dietary history questionnaire
ER: Estrogen receptors
FFQ: Food frequency questionnaire
HRCs: Hormone-related cancers
HRs: Hazard ratios
PRISMA: Preferred Reporting Items for Systematic Reviews and Meta-Analyses
NOS: Newcastle–Ottawa Scale
ORs: Odds ratios
RCTs: Randomized controlled trials
RRs: Relative risks
USDA: The U.S. Department of Agriculture
