# Supplementary material for: Consumption of flavonoids and risk of hormone-related cancers: a systematic review and meta-analysis of observational studies
Source: Nutr J. 2022 May 11;21:27. doi: 10.1186/s12937-022-00778-w (PMC9092883; doi:10.1186/s12937-022-00778-w)
Supplement: Supplementary file 1 — Additional file1. [file 12937_2022_778_MOESM1_ESM.docx]

**Supplementary Material**

**Supplementary Table S1**. Main characteristics of the eligible studies on flavonoid consumption and the risk of hormone-related cancers included in the meta-analysis.

**Supplementary Table S2**. Summary of Newcastle-Ottawa Scale (NOS) scores of cohort studies on flavonoid consumption and the risk of hormone-related cancers included in the meta-analysis.

**Supplementary Table S3**. Summary of Newcastle-Ottawa Scale (NOS) scores of case-control and nested case-control studies on flavonoid consumption and the risk of hormone-related cancers included in the meta-analysis.

**Supplementary Table S4**. Summary risk estimates and subgroup analyses based on study design for the association between flavonoid consumption and the risk of hormone-related cancers.

**Supplementary Table S5**. Subgroup analyses based on menopausal status for the association between flavonoid consumption and breast cancer risk.

**Supplementary Table S6**. Subgroup analyses based on study region for the association between flavonoid consumption and the risk of hormone-related cancers.

**Supplementary Table S7**. Begg's and Egger’s test detecting publication bias for included studies on flavonoid consumption and the risk of hormone-related cancers.

**Supplementary Table S8**. Sensitivity analyses for the consumption of total flavonoids in hormone-related cancers by sequential removal of each study.

**Supplementary Table S1**. Main characteristics of the included eligible studies on flavonoid consumption and the risk of hormone-related cancers in the meta-analysis.

| Author, year | Study design, follow-up (for cohort), region | No. of cases; cohort size/No. of controls | Gender, age | Exposure assessment | Flavonoids / Subclasses | Comparison^a^ | Adjustments | NOS Scores^b^ |  |
| --- | --- | --- | --- | --- | --- | --- | --- | --- | --- |
| *Breast* |  |  |  |  |  |  |  |  |  |
| Horn-Ross and colleagues, 2001 | Population-based case-control study, USA | 1,272/1,610 | F, 35-79 y | FFQ | Genistein  Daidzein  Biochanin A  Formononetin  Total isoflavones | Q4 (≥1,440 ug/d) vs. Q1 (<480 ug/d)  Q4 (≥1,223 ug/d) vs. Q1 (<473 ug/d)  Q4 (≥83 ug/d) vs. Q1 (<22 ug/d)  Q4 (≥40 ug/d) vs. Q1 (<9 ug/d)  Q4 (≥2,775 ug/d) vs. Q1 (<1,048 ug/d) | Age, race/ethnicity, age at menarche, parity, lactation, history of benign breast disease, family history of breast cancer, education, menopausal status, BMI, and HRT use, and daily caloric intake. | 8 |  |
| Knekt and colleagues, 2002 | Prospective cohort study, 1967-1996 (30y), Finland | 125/4,647 | F, 15-99 y | DHTQ | Quercetin  Kaempferol  Myricetin  Hesperetin  Naringenin  Total flavonoids | Q4 (>4.7 mg/d) vs. Q1 (<1.8 mg/d)  Q4 (>0.9 mg/d) vs. Q1 (<0.2 mg/d)  Q4 (>0.20 mg/d) vs. Q1 (<0.03 mg/d)  Q4 (>26.8 mg/d) vs. Q1 (<3.2 mg/d)  Q4 (>7.7 mg/d) vs. Q1 (<0.9 mg/d)  Q4 (>39.5 mg/d) vs. Q1 (<8.5 mg/d) | Sex, age, geographic area, occupation, smoking, BMI. | 8 |  |
| Linseisen and colleagues, 2004 | Population-based case-control study, Germany | 278/666 | F, 24-52 y | FFQ | Formononetin  Biochanin A  Daidzein  Genistein  Isoflavonoids | Q4 (>149.6 ug/d) vs. Q1 (<42.1 ug/d)  Q4 (>19.7 ug/d) vs. Q1 (<6.3 ug/d)  Q4 (>192.6 ug/d) vs. Q1 (<60.2 ug/d)  Q4 (>81.0 ug/d) vs. Q1 (<30.6 ug/d)  Q4 (>414.7 ug/d) vs. Q1 (<173.7 ug/d) | First-degree family history of breast cancer, number of births, duration of breast-feeding, energy intake, BMI, alcohol consumption, education. | 5 |  |
| Bosetti and colleagues, 2005 | Hospital-based case-control study, Italy | 2,569/2,588 | F, 20-74 y | FFQ | Flavanones  Flavan-3-ols  Flavonols  Flavones  Anthocyanidins  Isoflavones | Q5 (>62.2 mg/d) vs. Q1 (<11.5 mg/d)  Q5 (>79.7 mg/d) vs. Q1 (<18.1 mg/d)  Q5 (>29.9 mg/d) vs. Q1 (<12.6 mg/d)  Q5 (>0.6 mg/d) vs. Q1 (<0.2 mg/d)  Q5 (>20.5 mg/d) vs. Q1 (<3.7 mg/d)  Q5 (>34.7 ug/d) vs. Q1 (<13.4 ug/d) | Age, study center, education, parity, alcohol consumption, nonalcohol energy intake. | 6 |  |
| Adebamowo and colleagues, 2005 | Prospective cohort study, 1991-1999 (8y), USA | 710/90,638 | F, 25-46 y | FFQ | Total flavonols  Kaempferol  Quercetin  Myricetin | Q5 (43.8 mg/d) vs. Q1 (6.8 mg/d)  Q5 (12.9 mg/d) vs. Q1 (0.80 mg/d)  Q5 (30.1 mg/d) vs. Q1 (5.3 mg/d)  Q5 (2.62 mg/d) vs. Q1 (0.09 mg/d) | Age at menarche, parity and age at first birth, family history of breast cancer in mother and/or sister, history of benign breast disease, OC use, alcohol consumption, energy intake, current BMI, height, smoking habit, physical activity, menopausal status. | 7 |  |
| Fink and colleagues, 2007 | Population-based case-control study, USA | 1,434/1,440 | F, 25-98 y | FFQ | Total flavonoids  Flavonols  Flavones  Flavanones  Flavan-3-ols  Anthocyanidins  Isoflavones | Q5 (≥364.8 mg/d) vs. Q1 (<44.6 mg/d)  Q5 (≥16.3 mg/d) vs. Q1 (<4.0 mg/d)  Q5 (≥0.23 mg/d) vs. Q1 (<0.05 mg/d)  Q5 (≥50.4 mg/d) vs. Q1 (<4.5 mg/d)  Q5 (≥268.0 mg/d) vs. Q1 (<6.5 mg/d)  Q5 (≥4.58 mg/d) vs. Q1 (<0.04 mg/d)  Q5 (≥0.62 mg/d) vs. Q1 (<0.17 mg/d) | Age and energy intake. | 6 |  |
| Torres-Sanchez and colleagues, 2008 | Hospital-based case-control study, Mexico | 141/141 | F, 21-79 y | FFQ | Flavonols  Flavones  Flavan-3-ols | T3 (>36.8 mg/d) vs. T1 (<26.0 mg/d)  T3 (>4.0 mg/d) vs. T1 (<1.6 mg/d)  T3 (>10.6 mg/d) vs. T1 (<5.9 mg/d) | Age, total energy consumption, lifetime lactation, menopausal stage. | 5 |  |
| Touvier and colleagues, 2013 | Prospective cohort study, 1994-2007 (median 12.6 y), France | 152/4,141 | F, 47 y  (mean) | FFQ | Flavonoids  Anthocyanins  Flavonols  Flavanones  Flavones  Catechins  Theaflavins | Q4 (631.7 mg/d) vs. Q1 (294.2 mg/d)  Q4 (56.9 mg/d) vs. Q1 (24.5 mg/d)  Q4 (59.8 mg/d) vs. Q1 (33.0 mg/d)  Q4 (28.3 mg/d) vs. Q1 (18.6 mg/d)  Q4 (30.9 mg/d) vs. Q1 (23.5 mg/d)  Q4 (151.5 mg/d) vs. Q1 (61.2 mg/d)  Q4 (21.2 mg/d) vs. Q1 (8.1 mg/d) | Age, BMI, intervention group, alcohol intake, number of dietary records, and energy intake, height, physical activity, smoking status, educational level, family history of breast cancer, menopausal status, use of hormonal treatment for menopause, number of children. | 9 |  |
| Zamora-Ros and colleagues, 2013 | Multicentre prospective cohort study, 1992-2010 (median 11.5 y), Europe | 11,576/334,850 | F, 35-70 y | FFQ | Total flavonoids  Flavanols  Flavan-3-ol monomers  Theaflavins  Anthocyanidins  Flavonols  Flavanones  Flavones  Isoflavones | Q5 (>654.0 mg/d) vs. Q1 (<176.0 mg/d)  Q5 (>550.5 mg/d) vs. Q1 (<121.2 mg/d)  Q5 (>379.8 mg/d) vs. Q1 (<18.2 mg/d)  Q5 (>13.88 mg/d) vs. Q1 (0 mg/d)  Q5 (>43.6 mg/d) vs. Q1 (<12.1 mg/d)  Q5 (>39.8 mg/d) vs. Q1 (<12.8 mg/d)  Q5 (>33.0 mg/d) vs. Q1 (<6.2 mg/d)  Q5 (>4.88 mg/d) vs. Q1 (<1.12 mg/d)  Q5 (>1.36 mg/d) vs. Q1 (<0.22 mg/d) | Centre, age, baseline menopausal status, weight, height, smoking status, educational level, physical activity, age at menarche, age at first full-term birth, ever use of contraceptive pills, ever use of hormones, age at menopause, energy intake, alcohol intake, fiber intake. | 8 |  |
| Wang and colleagues, 2014 | Prospective cohort study, 1999-2009 (median 8.5 y), USA | 2,116/56,630 | F, ~68 y  (mean) | FFQ | Total flavonoids  Anthocyanidins  Flavan-3-ols  Flavanones  Flavones  Flavonols  Isoflavones | Q5 (>364 mg/d) vs. Q1 (≤119 mg/d)  Q5 (>16.1 mg/d) vs. Q1 (≤5.3 mg/d)  Q5 (>36.7 mg/d) vs. Q1 (≤9.0 mg/d)  Q5 (>34.0 mg/d) vs. Q1 (≤6.5 mg/d)  Q5 (>2.1 mg/d) vs. Q1 (≤0.6 mg/d)  Q5 (>20.8 mg/d) vs. Q1 (≤8.3 mg/d)  Q5 (>0.093 mg/d) vs. Q1 (≤0.026 mg/d) | Age, family history of breast cancer, history of breast cyst, weight gain since age 18 y, education, combination of parity and age at first live birth, age at menopause, ethanol consumption, smoking history, TEI in quintile, and history of HRT. | 9 |  |
| Wang and colleagues, 2009 | Prospective cohort study, 1992-2007 (median 11.5 y), USA | 1,351/38,408 | F, ≥45 y | SFFQ | Total flavonoids | Q5 (≥34.55 mg/d) vs. Q1 (≤11.55 mg/d) | Age, race, TEI, randomized treatment assignment, smoking, alcohol use, physical activity, postmenopausal status, HRT use, multivitamin use, BMI, family history of colorectal cancer, ovarian cancer, and breast cancer, intake of fruit and vegetables, fiber, folate, and saturated fat, age at menarche, number of pregnancies lasting ≥6 m, age at first pregnancy lasting ≥6 m, and OC use. | 8 |  |
| Li and colleagues, 2013 | Population-based case-control study, China | 560/560 | F, 18-85 y | FFQ | Daidzein  Genistein  Glycitein  Total isoflavone | Q4 (>19.47 mg/d) vs. Q1 (<6.33 mg/d)  Q4 (>25.44 mg/d) vs. Q1 (<8.46 mg/d)  Q4 (>1.46 mg/d) vs. Q1 (<0.38 mg/d)  Q4 (>35.12 mg/d) vs. Q1 (<12.49 mg/d) | Education, income, BMI, smoking, passive smoking, alcohol consumption, green tea drinking, physical activity, TEI, cancer in first-degree relative. | 5 |  |
| Luo and colleagues, 2010 | nested case-control study, (1997–2006, median 6.1 y), China | 352/701 | F, 40-70 y | Urinary Samples | Flavanols  Flavonols  Kaempferol  Quercetin | T3 vs. T1  T3 vs. T1  T3 vs. T1  T3 vs. T1 | Age, education, age at menarche, age at 1st live birth, months of breast fibroadenoma, first-degree family cancer history, ever smoke, total intake of red meat and isoflavones, use of HRT and tea intakes. | 9 |  |
| Dai and colleagues, 2002 | Population-based case-control study, China | 250/250 | F, 25-64 y | Urinary Samples | Total isoflavonoids  Daidzein  Genistein  Glycitein  Flavanones  Hesperetin  Naringenin | T3 vs. T1  T3 vs. T1  T3 vs. T1  T3 vs. T1  T3 vs. T1  T3 vs. T1  T3 vs. T1 | Age at first live birth, ever diagnosed with fibroadenoma, total meat intake, and physical activity level. | 7 |  |
| Kyrø and colleagues, 2015 | Prospective cohort study, 1993-2010 (median 6 y), Europe | 753/11,782 | F, 60 y (mean) | CSDQ | Flavonoids  Flavanols  Anthocyanins  Flavanones  Flavones  Flavonols  Isoflavonoids | Q4 (>705 mg/d) vs. Q1(≤271 mg/d)  Q4 vs. Q1  Q4 vs. Q1  Q4 vs. Q1  Q4 vs. Q1  Q4 vs. Q1  Q4 vs. Q1 | For flavonoids: Alcohol, BMI, HRT use, Schooling, smoking status, physical activity index;  For others: Alcohol, BMI, HRT use, Schooling, smoking status, physical activity index, ER status, cancer stage, grading of tumor. | 7 |  |
| Pantavos and colleagues, 2015 | Prospective cohort study, 1989-2010 (median 17 y), Netherlands | 199/3,209 | F, ≥55 y | SFFQ | Flavonoids | T3 (40.46 mg/d) vs. T1 (18.07 mg/d) | Age, BMI, educational level, family history of breast cancer, smoking status, alcohol consumption, and use of multivitamin supplement. | 8 |  |
| Fink and colleagues, 2007 | Prospective cohort study, 1996-2002 (6 y), USA | 113/1,210 | F, 25-98 y | FFQ | Total flavonoids  Flavonols  Flavones  Flavanones  Flavan-3-ols  Anthocyanidins  Isoflavones | Q5 (≥340.5 mg/d) vs. Q1 (≤42.4 mg/d)  Q5 (≥14.5 mg/d) vs. Q1 (≤3.4 mg/d)  Q5 (≥0.20 mg/d) vs. Q1 (≤0.03 mg/d)  Q5 (≥48.6 mg/d) vs. Q1 (≤4.0 mg/d)  Q5 (≥263.8 mg/d) vs. Q1 (≤5.0 mg/d)  Q5 (≥4.24 mg/d) vs. Q1 (≤0.03 mg/d)  Q5 (≥0.60 mg/d) vs. Q1 (≤0.15 mg/d) | Age and energy intake. | 8 |  |
| Zheng and colleagues, 1999 | Population-based case-control study, China | 60/60 | F, 25-64 y | Spot Urine Samples | Total isoflavonoids  Daidzein  Genistein  Glycitein | T3 (≥18.66 nmol/mg) vs. T1 (<5.58 nmol/mg)  T3 (≥7.61 nmol/mg) vs. T1 (<2.81 nmol/mg)  T3 (≥4.09 nmol/mg) vs. T1 (<0.98 nmol/mg)  T3 (≥2.15 nmol/mg) vs. T1 (<0.63 nmol/mg) | Age at first pregnancy and physical activity levels. | 7 |  |
| Feng and colleagues, 2019 | Hospital-based case-control study, China | 1,522/1,547 | F, 25-70 y | FFQ | Total flavonoids  Anthocyanidins  Flavanols  Flavan-3-ol monomers  Theaflavins  Flavanones  Flavones  Flavonols  Isoflavones | Q4 vs. Q1  Q4 vs. Q1  Q4 vs. Q1  Q4 vs. Q1  Q4 vs. Q1  Q4 vs. Q1  Q4 vs. Q1  Q4 vs. Q1  Q4 vs. Q1 | Age, BMI, education, income, passive smoking, physical activity, alcohol intake, age at menarche, first-degree relative of cancer, history of benign breast disease, and ever use of OC. | 7 |  |
| Feng and colleagues, 2021 | Hospital-based case-control study, China | 792/813 | F, 25-70 y | Serum | Flavonols  Quercetin  Isohamnetin  Kaempferol  Flavones  Apigenin  Luteolin  Flavanones  Naringenin  Hesperetin  Flavan-3-ols  Catechin  Epicatechin  Epigallocatechin  Epicatechin-3-gallate  Epigallocatechin-3-gallate | Q4 (>17.06 ng/mL) vs. Q1 (<3.94 ng/mL)  Q4 (>6.13 ng/mL) vs. Q1 (<1.39 ng/mL)  Q4 (>4.56 ng/mL) vs. Q1 (<0.92 ng/mL)  Q4 (>6.11 ng/mL) vs. Q1 (<0.70 ng/mL)  >LOD vs. <LOD  >LOD vs. <LOD  >LOD vs. <LOD  Q4 (>2.26 ng/mL) vs. Q1 (<0.18 ng/mL)  Q4 (>0.90 ng/mL) vs. Q1 (<0.10 ng/mL)  Q4 (>1.25 ng/mL) vs. Q1 (<0.01 ng/mL)  Q4 (>5.60 ng/mL) vs. Q1 (<0.29 ng/mL)  >LOD vs. <LOD  >LOD vs. <LOD  >LOD vs. <LOD  Q4 (>3.24 ng/mL) vs. Q1 (0.00 ng/mL)  Q4 (>1.07 ng/mL) vs. Q1 (0.00 ng/mL) | Age, education, BMI, MET-hours/wk, passive smoking, alcohol intake, fiber intake, age at menarche, history of benign breast disease, and first-degree relative with cancer. | 8 |  |
| Feng and colleagues, 2021 |  |  |  |  | Total soy isoflavones  Daidzein  Genistein  Glycitein  Formononetin | Q4 (>29.25 ng/mL) vs. Q1 (<2.6 ng/mL)  Q4 (>6.75 ng/mL) vs. Q1 (<0.73 ng/mL)  Q4 (>19.92 ng/mL) vs. Q1 (<1.5 ng/mL)  Q4 (>1.11 ng/mL) vs. Q1 (0.00 ng/mL)  high (>0.02 ng/mL) vs. <LOD | Age, education, BMI, MET-hours/wk, passive smoking, alcohol intake, age at menarche, history of benign breast disease, and first-degree relative with cancer. |  |  |
| *Endometrium* |  |  |  |  |  |  |  |  |  |
| Horn-Ross and colleagues, 2013 | Population-based case-control study, USA | 500/470 | F, 35-79 y | FFQ | Genistein  Daidzein  Biochanin A  Formononetin  Total isoflavones | Q4 (≥1,420 ug/d) vs. Q1 (<500 ug/d)  Q4 (≥1,197 ug/d) vs. Q1 (<521 ug/d)  Q4 (≥81 ug/d) vs. Q1 (<21 ug/d)  Q4 (≥36 ug/d) vs. Q1 (<10 ug/d)  Q4 (≥2,726 ug/d) vs. Q1 (<1,150 ug/d) | Age, race/ethnicity, daily caloric intake, age at menarche, parity, use of OC and HRT, BMI. | 7 |  |
| Xu and colleagues, 2008 | Population-based case-control study, China | 1,199/1,212 | F, 30-69 y | FFQ | Soy isoflavone | T3 (>40.3 mg/d) vs. T1 (≤21.3 mg/d) | Age, education, menopausal status, years of menstruation, number of pregnancies, OC use, alcohol consumption, diagnosis of diabetes, BMI, physical activity, caloric intake. | 6 |  |
| Bandera and colleagues, 2009 | Population-based case-control study, USA | 424/398 | F, 63 y (mean) | FFQ | Total isoflavones  Daidzein  Genistein  Formononetin  Glycitein  Quercetin | Q4 (>666.0 ug/1000 Kcal/d) vs. Q1 (<55.3 ug/1000 Kcal/d)  Q4 (>269.9 ug/1000 Kcal/d) vs. Q1 (<15.7 ug/1000 Kcal/d)  Q4 (>369.6 ug/1000 Kcal/d) vs. Q1 (<30.5 ug/1000 Kcal/d)  Q4 (≥8.5 ug/1000 Kcal/d) vs. Q1 (<3.4 ug/1000 Kcal/d)  Q4 (>19.3 ug/1000 Kcal/d) vs. Q1 (<1.9 ug/1000 Kcal/d)  Q4 (>1507.8 ug/1000 Kcal/d) vs. Q1 (<1083.6 ug/1000 Kcal/d) | Age, education, race, age at menarche, menopausal status and age at menopause for postmenopausal women, parity, OC use, HRT use, BMI, total calories, physical activity, smoking status, alcohol. | 7 |  |
| Ollberding and colleagues, 2012 | Prospective cohort study, 1993-2007 (13.6 y), USA | 489/46,027 | F, 45-75 y | QFFQ | Total isoflavones  Daidzein  Genistein  Glycitein | Q5 (11.23 mg/1000 Kcal/d) vs. Q1 (0.87 mg/1000 Kcal/d)  Q5 (5.09 mg/1000 Kcal/d) vs. Q1 (0.38 mg/1000 Kcal/d)  Q5 (4.87 mg/1000 Kcal/d) vs. Q1 (0.38 mg/1000 Kcal/d)  Q5 (1.28 mg/1000 Kcal/d) vs. Q1 (0.1 mg/1000 Kcal/d) | Age, race or ethnicity, and age at cohort entry, BMI, age at menarche, age at menopause, duration and type of HRT use, duration of OC use, parity, smoking status, hypertension, diabetes, total calories. | 8 |  |
| Rossi and colleagues, 2013 | Hospital-based case-control study, Italy | 454/908 | F, 18-80 y | FFQ | Flavanols  Flavanones  Flavonols  Anthocyanidins  Flavones  Isoflavones | Q4 (>69.1 mg/d) vs. Q1-Q3 (<69.1 mg/d)  Q4 (>54.7 mg/d) vs. Q1-Q3 (<54.7 mg/d)  Q4 (>24.7 mg/d) vs. Q1-Q3 (<24.7 mg/d)  Q4 (>15.6 mg/d) vs. Q1-Q3 (<15.6 mg/d)  Q4 (>0.6 mg/d) vs. Q1-Q3 (<0.6 mg/d)  Q4 (>58.2 ug/d) vs. Q1-Q3 (<58.2 ug/d) | Study centre, age, year of interview, education, BMI, history of diabetes, age at menarche, menopausal status/age at menopausal status, parity, OC use, HRT use. | 6 |  |
| Neill and colleagues, 2014 | Population-based case-control study, Australia | 1,288/1,435 | F, 18-79 y | FFQ | Isoflavones  Daidzein  Genistein  Glycitein  Biochanin A  Formononetin | Q4 (>4,000 ug/d) vs. Q1 (<280 ug/d)  Q4 (>1,200 ug/d) vs. Q1 (<90 ug/d)  Q4 (>2,700 ug/d) vs. Q1 (<150 ug/d)  Q4 (>250 ug/d) vs. Q1 (<20 ug/d)  Q4 (>30 ug/d) vs. Q1 (<15 ug/d)  Q4 (>5.0 ug/d) vs. Q1 (<2.5 ug/d) | Age, energy intake (log transformed), hysterectomy, menopausal status, education, parity, OC use, HRT use, BMI, alcohol consumption and smoking status. | 8 |  |
| Budhathoki and colleagues, 2015 | Prospective cohort study, 1990-2009 (average 12.1 y), Japan | 112/49,121 | F, 40-69 y | FFQ | Isoflavone | T3 (63.2 mg/d) vs. T1 (17.7 mg/d) | Age, PHC-area, BMI, physical activity, smoking, alcohol consumption, age at menarche, exogenous hormone use, number of deliveries, menopausal status and age at menopause for postmenopausal women, coffee intake, past history of diabetes mellitus and cancer. | 9 |  |
| Wang and colleagues, 2009 | Prospective cohort study, 1992-2007 (median 11.5 y), USA | 259/38,408 | F, ≥45 y | SFFQ | Total flavonoids | Q5 (≥34.55 mg/d) vs. Q1 (≤11.55 mg/d) | Age, race, TEI, randomized treatment assignment , smoking, alcohol use, physical activity, postmenopausal status , HRT use, multivitamin use, BMI, family history of colorectal cancer, ovarian cancer, and breast cancer, intake of fruit and vegetables, fiber, folate, and saturated fat, age at menarche, number of pregnancies lasting ≥6 m, age at first pregnancy lasting ≥6 m, and OC use. | 8 |  |
| *Ovary* |  |  |  |  |  |  |  |  |  |
| Chang and colleagues, 2007 | Prospective cohort study, 1995-2003 (median 8.1 y), USA | 280/97,275 | F, 20-84 y | FFQ | Total isoflavones  Genistein  Daidzein | T3 (>3 mg/d) vs. T1 (<1 mg/d)  Q5 (>1.1 mg/d) vs. Q1 (≤0.3 mg/d)  Q5 (>0.9 mg/d) vs. Q1 (≤0.3 mg/d) | Race, TEI, parity, OC use, strenuous exercise, wine consumption, menopausal status/hormone therapy use, age at baseline. | 6 |  |
| Gates and colleagues, 2007 | Prospective cohort study, 1984-2002 (18 y), USA | 347/66,940 | F, 30-55 y | FFQ | Total flavonoids  Myricetin  Kaempferol  Quercetin  Luteolin  Apigenin | Q5 (42.6 mg/d) vs. Q1 (8.5 mg/d)  Q5 (2.4 mg/d) vs. Q1 (0.1 mg/d)  Q5 (11.0 mg/d) vs. Q1 (0.8 mg/d)  Q5 (30.7 mg/d) vs. Q1 (6.3 mg/d)  Q5 (0.07 mg/d) vs. Q1 (0.01 mg/d)  Q5 (1.3 mg/d) vs. Q1 (0.2 mg/d) | Age, duration of OC use, parity, history of tubal ligation, smoking status, history of postmenopausal hormone use, physical activity, quintile of cumulative updated energy-adjusted lactose intake and cumulative updated TEI, quintile of intake of each other individual flavonoid. | 7 |  |
| Rossi and colleagues, 2008 | Hospital-based case-control study, Italy | 1,031/2,411 | F, 18-79 y | FFQ | Flavan-3-ols  Flavanones  Flavonols  Anthocyanidins  Flavones  Isoflavones  Total flavonoids | Q5 (>77.0 mg/d) vs. Q1 (<16.3 mg/d)  Q5 (>67.0 mg/d) vs. Q1 (<12.2 mg/d)  Q5 (>28.8 mg/d) vs. Q1 (<11.6 mg/d)  Q5 (>19.4 mg/d) vs. Q1 (<3.5 mg/d)  Q5 (>0.7 mg/d) vs. Q1 (<0.3 mg/d)  Q5 (>32.5 ug/d) vs. Q1 (<12.8 ug/d)  Q5 (>173.6 mg/d) vs. Q1 (<67.3 mg/d) | Age, study center, education, year of interview, parity, OC use, family history of ovarian cancer or breast cancer or both in first-degree relatives. | 7 |  |
| Gates and colleagues, 2009 | Population-based case-control study, USA | 1,141/1,183 | F, 51 y (mean) | FFQ | Total flavonoid  Myricetin  Kaempferol  Quercetin  Luteolin  Apigenin | Q5 (27.5 mg/d) vs. Q1 (6.0 mg/d)  Q5 (2.8 mg/d) vs. Q1 (0.4 mg/d)  Q5 (6.9 mg/d) vs. Q1 (0.5 mg/d)  Q5 (16.5 mg/d) vs. Q1 (3.5 mg/d)  Q5 (2.9 mg/d) vs. Q1 (0.3 mg/d)  Q5 (0.7 mg/d) vs. Q1 (0.03 mg/d) | Age in years, study center, duration of OC use, parity, history of tubal ligation, physical activity, total duration of breastfeeding, dietary intake of carotenoids, fiber intake, and TEI, quintile of intake of each other individual flavonoid. | 8 |  |
| Bandera and colleagues, 2011 | Population-based case-control study, USA | 205/390 | F, >55 y | FFQ | Daidzein  Genistein  Formononetin  Glycitein  Total isoflavones | T3 (≥144.08 ug/1000 Kcal/d) vs. T1 (<20.25 ug/1000 Kcal/d)  T3 (≥247.86 ug/1000 Kcal/d) vs. T1 (<40.46 ug/1000 Kcal/d)  T3 (≥6.81 ug/1000 Kcal/d) vs. T1 (<3.90 ug/1000 Kcal/d)  T3 (≥9.18 ug/1000 Kcal/d) vs. T1 (<2.14 ug/1000 Kcal/d)  T3 (≥404.67 ug/1000 Kcal/d) vs. T1 (<70.06 ug/1000 Kcal/d) | Age, education, race, age at menarche, menopausal status, parity, OC use, HRT use, BMI, tubal ligation, total calories, physical activity, smoking, alcohol intake. | 8 |  |
| Hedelin and colleagues, 2011 | Prospective cohort study, 1991-2007 (16 y), Sweden | 163/47,140 | F, 30-49 y | FFQ | Total isoflavonoids | Q4 (38 ug/d*MJ) vs. Q1 (0.5 ug/d*MJ) | Age, OC, age at menarche, parity, HRT, and intake of TEI, alcohol, saturated fat, meat, and fish. | 8 |  |
| Cassidy and colleagues, 2014 | 2 Prospective cohort study (NHS 1984-2010, NHSII 1991-2011), (16-22 y), USA | 723/171,940  (82,289 NHS,  89,651 NHSII) | NHS  F, 30-55 y  NHSII  F, 25-42 y | FFQ | Total flavonoids  Flavonols  Flavones  Flavanones  Flavan-3-ols  Anthocyanidins | Q5 (713.4 mg/d) vs. Q1 (117.1 mg/d)  Q5 (30.2 mg/d) vs. Q1 (7.4 mg/d)  Q5 (3.2 mg/d) vs. Q1 (0.7 mg/d)  Q5 (75.8 mg/d) vs. Q1 (7.8 mg/d)  Q5 (133.7 mg/d) vs. Q1 (9.3 mg/d)  Q5 (23.9 mg/d) vs. Q1 (2.5 mg/d) | Age, calendar time, menopausal status, duration of OC use, parity, history of tubal ligation, history of hysterectomy, duration of postmenopausal hormone use by type, family history of breast cancer or ovarian cancer, quintiles of cumulative updated energy-adjusted lactose intake and caffeine intake, quintiles of cumulative updated TEI. | 8 |  |
| Lee and colleagues, 2014 | Hospital-based case-control study, China | 500/500 | F, 59 y  (average) | FFQ | Isoflavones  Daidzein  Genistein  Glycitein | T3 (>41.0 mg/d) vs. T1 (≤26.7 mg/d)  T3 (>16.9 mg/d) vs. T1 (≤10.2 mg/d)  T3 (>21.1 mg/d) vs. T1 (≤12.3 mg/d)  T3 (>3.3 mg/d) vs. T1 (≤1.9 mg/d) | Age, BMI, physical activity, TEI, parity, OC use, HRT, menopausal status, education, smoking status, alcohol drinking, family history of ovarian cancer or breast cancer. | 8 |  |
| Neill and colleagues, 2014 | Population-based case-control study, Australia | 1,366/1,414 | F, 18-79 y | SFFQ | Isoflavones  Daidzein  Genistein  Glycitein  Biochanin A  Formononetin | Q4 (>4,000 ug/d) vs. Q1 (<280 ug/d)  Q4 (>1,200 ug/d) vs. Q1 (<90 ug/d)  Q4 (>2,700 ug/d) vs. Q1 (<150 ug/d)  Q4 (>250 ug/d) vs. Q1 (<20 ug/d)  Q4 (>30 ug/d) vs. Q1 (<15 ug/d)  Q4 (>5.0 ug/d) vs. Q1 (<2.5 ug/d) | Age, energy intake (log transformed), hysterectomy, menopausal status, education, parity, OC use, HRT use, BMI, alcohol consumption and smoking status. | 8 |  |
| Wang and colleagues, 2009 | Prospective cohort study, 1992-2007 (median 11.5 y), USA | 141/38,408 | F, ≥45 y | SFFQ | Total flavonoids | Q5 (≥34.55 mg/d) vs. Q1 (≤11.55 mg/d) | Age, race, TEI, randomized treatment assignment , smoking, alcohol use, physical activity, postmenopausal status , HRT use, multivitamin use, BMI, family history of colorectal cancer, ovarian cancer, and breast cancer, intake of fruit and vegetables, fiber, folate, and saturated fat, age at menarche, number of pregnancies lasting ≥6 m, age at first pregnancy lasting ≥6 m, and OC use. | 8 |  |
| Zhang and colleagues, 2004 | Hospital-based case-control study, China | 254/652 | F, < 75 y | FFQ | Total isoflavones  Daidzein  Genistein  Glycitein | Q4 (≥32.8 mg/d) vs. Q1 (≤11.6 mg/d)  Q4 (≥14.9 mg/d) vs. Q1 (≤5.0 mg/d)  Q4 (≥20.9 mg/d) vs. Q1 (≤6.6 mg/d)  Q4 (≥1.7 mg/d) vs. Q1 (≤0.4 mg/d) | Age at diagnosis, education, area of residence, BMI, tobacco smoking, alcohol consumption, tea drinking, physical activity, age at menarche, parity, menopausal status, HRT, OC use, ovarian cancer in first-degree relatives, TEI. | 6 |  |
| *Prostate* |  |  |  |  |  |  |  |  |  |
| Knekt and colleagues, 2002 | Prospective cohort study, 1967-1996 (30y), Finland | 95/5,218 | F, 15-99 y | DHTQ | Quercetin  Kaempferol  Myricetin  Hesperetin  Naringenin  Total flavonoids | Q4 (>4.7 mg/d) vs. Q1 (<1.8 mg/d)  Q4 (>0.9 mg/d) vs. Q1 (<0.2 mg/d)  Q4 (>0.20 mg/d) vs. Q1 (<0.03 mg/d)  Q4 (>26.8 mg/d) vs. Q1 (<3.2 mg/d)  Q4 (>7.7 mg/d) vs. Q1 (<0.9 mg/d)  Q4 (>39.5 mg/d) vs. Q1 (<8.5 mg/d) | Sex, age, geographic area, occupation, smoking, BMI. | 8 |  |
| Bosetti and colleagues, 2006 | Hospital-based case-control study, Italy | 1,294/1,451 | M, 46-74 y | FFQ | Flavanones  Flavan-3-ols  Flavonols  Flavones  Anthocyanidins  Isoflavones  Total flavonoids | Q5 (>46.9 mg/d) vs. Q1 (≤5.2 mg/d)  Q5 (>102.1 mg/d) vs. Q1 (≤29.9 mg/d)  Q5 (>30.7 mg/d) vs. Q1 (≤15.1 mg/d)  Q5 (>0.6 mg/d) vs. Q1 (≤0.2 mg/d)  Q5 (>40.3 mg/d) vs. Q1 (≤8.3 mg/d)  Q5 (>32.2 ug/d) vs. Q1 (≤14.7 ug/d)  Q5 (>240.8 mg/d) vs. Q1 (≤109.4 mg/d) | Age, study center, education, BMI, family history of prostate cancer, total calorie intake. | 5 |  |
| Hedelin and colleagues, 2006 | Population-based case-control study, Sweden | 1,314/782  1,499/1,130 | M, 35-79 y | FFQ | Isoflavonoids  Genistein  Daidzein | Q4 (113 ug/d*MJ) vs. Q1 (0.8 ug/d*MJ)  Q4 (69.4 ug/d*MJ) vs. Q1 (0.19 ug/d*MJ)  Q4 (43.1 ug/d*MJ) vs. Q1 (0.37 ug/d*MJ) | Age and intake of antibiotics, zinc, animal fat, TEI, alcohol, vegetable fat, and red meat (and total lignans for genistein and daidzein) during the last year. | 6 |  |
| Heald and colleagues, 2007 | Population-based case-control study, Britain | 433/483 | M, 50-74 y | FFQ;  Serum Samples | Isoflavone  Daidzein  Genistein | Q4 (>1,982.8 ug/d) vs. Q1 (<581.1 ug/d)  Q4 (>29.11 nmol/L) vs. Q1 (<8.26 nmol/L)  Q4 (>64.53 nmol/L) vs. Q1 (<14.23 nmol/L) | Age, TEI, family history of prostate cancer and breast cancer, Carstairs Deprivation Index, smoking status and energy intake: BMR ratio. | 7 |  |
| Nagata and colleagues, 2007 | Hospital-based case-control study, Japan | 200/200 | M, 59-73 y | FFQ | Isoflavones  Genistein  Daidzein | Q4 (≥89.9 mg/d) vs. Q1 (<30.5 mg/d)  Q4 (≥2.5 mg/d) vs. Q1 (<1.1 mg/d)  Q4 (≥1.9 mg/d) vs. Q1 (<0.8 mg/d) | Cigarette smoking and energy and PUFA intakes. | 5 |  |
| Mursu and colleagues, 2008 | Prospective cohort study, 1984-2005 (mean 16.2 y), Finland | 138/2,590 | M, 53 y  (mean) | 4-DFR | Flavonols  Flavones  Flavanones  Flavan-3-ols  Anthocyanidins  Total flavonoids | Q4 vs. Q1  Q4 vs. Q1  Q4 vs. Q1  Q4 vs. Q1  Q4 vs. Q1  Q4 (415.8 mg/d) vs. Q1 (0 mg/d) | Age, examination years, BMI, smoking status, pack-years of smoking, physical activity, intakes of alcohol, total fat, and saturated fat, energy adjusted intake of fiber, vitamin C and E. | 9 |  |
| Park and colleagues, 2008 | Prospective cohort study, 1993-2002 (9 y), USA | 4,404/82,483 | M, 45-75 y | QFFQ | Genistein  Daidzein  Glycitein  Total isoflavones | Q5 (≥3.1 mg/1000 Kcal/d) vs. Q1 (<0.7 mg/1000 Kcal/d)  Q5 (≥3.2 mg/1000 Kcal/d) vs. Q1 (<0.7 mg/1000 Kcal/d)  Q5 (≥0.80 mg/1000 Kcal/d) vs. Q1 (<0.18 mg/1000 Kcal/d)  Q5 (≥7.2 mg/1000 Kcal/d) vs. Q1 (<1.6 mg/1000 Kcal/d) | Time since cohort entry, ethnicity, family history of prostate cancer, education, BMI, smoking status, energy intake. | 8 |  |
| Geybels and colleagues, 2013 | Prospective cohort study, 1986-2003 (17.3 y), Netherlands | 3,362/58,279 | M, 55-69 y | SFFQ | Total catechin  Total flavonol  Quercetin  Kaempferol  Myricetin | Q4 (98.7 mg/d) vs. Q1 (14.5 mg/d)  Q4 (40.6 mg/d) vs. Q1 (13.1 mg/d)  Q4 (28.5 mg/d) vs. Q1 (8.8 mg/d)  Q4 (11.2 mg/d) vs. Q1 (2.5 mg/d)  Q4 (2.6 mg/d) vs. Q1 (0.4 mg/d) | Age. | 8 |  |
| Wang and colleagues, 2014 | Prospective cohort study, 1992-2009 (mean 7.8 y), USA | 3,974/43,268 | M, 50-74 y | SFFQ | Total flavonoids  Anthocyanidins  Flavan-3-ols  Flavanones  Flavones  Flavonols  Isoflavones | Q5 (504.8 mg/d) vs. Q1 (99.0 mg/d)  Q5 (23.8 mg/d) vs. Q1 (4.1 mg/d)  Q5 (63.9 mg/d) vs. Q1 (7.6 mg/d)  Q5 (53.9 mg/d) vs. Q1 (3.8 mg/d)  Q5 (3.2 mg/d) vs. Q1 (0.4 mg/d)  Q5 (26.6 mg/d) vs. Q1 (7.2 mg/d)  Q5 (0.72 mg/d) vs. Q1 (0.026 mg/d) | Age, race, family history of prostate cancer, BMI, smoking status, aspirin use, TEI, history of prostate-specific antigen screening, history of diabetes. | 9 |  |
| Reale and colleagues, 2018 | Population-based case-control study, Italy | 118/222 | M, 69 y (mean) | FFQ | Total flavonoids  Anthocyanins  Flavonols  Flavanols  Flavanones  Flavones  Catechins | Q4 vs. Q1  Q4 vs. Q1  Q4 vs. Q1  Q4 vs. Q1  Q4 vs. Q1  Q4 vs. Q1  Q4 vs. Q1 | Age, TEI, weight status, smoking status, alcohol consumption, physical activity level, and family history of prostate cancer. | 8 |  |
| Russo and colleagues, 2018 |  |  |  |  | Isoflavones  Daidzein  Genistein  Glycitein  Biochanin A | Q4 vs. Q1  Q4 vs. Q1  Q4 vs. Q1  high vs. low  T3 vs. T1 |  |  |  |
| Reger and colleagues, 2018 | Prospective cohort study, 1993-2009 (median 11.5 y), USA | 2,598/27,004 | M, 62 y  (mean) | FFQ | Total isoflavones  Genistein  Daidzein  Glycitein  Formononetin  Biochanin A | Q5 (≥0.75 mg/d) vs. Q1 (≤0.17 mg/d)  Q5 (≥0.35 mg/d) vs. Q1 (≤0.04 mg/d)  Q5 (≥0.41 mg/d) vs. Q1 (≤0.11 mg/d)  Q5 (≥0.027 mg/d) vs. Q1 (≤0.001 mg/d)  Q5 (≥0.022 mg/d) vs. Q1 (≤0.005 mg/d)  Q5 (≥0.087 mg/d) vs. Q1 (≤0.028 mg/d) | Age, race/ethnicity, BMI, smoking status, alcohol intake and family history of prostate cancer. | 8 |  |
| Ghanavati and colleagues, 2020 | Hospital-based case-control study, Iran | 97/205 | M, 40-80 y | SFFQ | Total flavonoids  Anthocyanins  Flavonols  Flavan-3-ols  Flavanones  Flavones | T3 (>1083.67 mg/d) vs. T1 (<718.29 mg/d)  T3 (>54.30 mg/d) vs. T1 (<23.17 mg/d)  T3 (>790.42 mg/d) vs. T1 (<438.74 mg/d)  T3 (>150.21 mg/d) vs. T1 (<94.27 mg/d)  T3 (>97.29 mg/d) vs. T1 (<58.33 mg/d)  T3 (>6.50 mg/d) vs. T1 (<3.35 mg/d) | Energy intake, hypertension, diabetes, smoking, BMI and waist circumstance. | 7 |  |
| *Testis* |  |  |  |  |  |  |  |  |  |
| Walcott and colleagues, 2002 | Population-based case-control study, USA | 187/148 | M, 18-55 y | FFQ | Flavonoids  Isoflavonoids | Q4 (>9.5 mg/1000 Kcal/d) vs. Q1 (<4.0 mg/1000 Kcal/d)  Q4 (>474.0 ug/1000 Kcal/d) vs. Q1 (<30.6 ug/1000 Kcal/d) | Ethnicity, age, education, income, history of cryptorchidism, early onset of puberty, history of severe adolescent acne, balding unrelated to cancer therapy, BMI, TEI, total fat intake, total dietary fiber intake, and phytoestrogen classes. | 8 |  |
| *Thyroid* |  |  |  |  |  |  |  |  |  |
| Xiao and colleagues, 2014 | Prospective cohort study, 1995-2006 (mean 9 y), USA | 586/491,840 | M&F,  50-71 y | FFQ | Flavan-3-ols  Flavanones  Flavonols  Anthocyanidins  Flavones  Isoflavones  Total flavonoids | Q5 (330.58 mg/1000 cal/d) vs. Q1 (8.58 mg/1000 cal/d)  Q5 (45.95 mg/1000 cal/d) vs. Q1 (2.65 mg/1000 cal/d)  Q5 (21.15 mg/1000 cal/d) vs. Q1 (4.66 mg/1000 cal/d)  Q5 (15.16 mg/1000 cal/d) vs. Q1 (2.12 mg/1000 cal/d)  Q5 (1.57 mg/1000 cal/d) vs. Q1 (0.18 mg/1000 cal/d)  Q5 (0.67 mg/1000 cal/d) vs. Q1 (0.1 mg/1000 cal/d)  Q5 (387.33 mg/1000 cal/d) vs. Q1 (34.29 mg/1000 cal/d) | Sex, total caloric intake, smoking status, education level, alcohol intake, race, BMI, family history of cancer. | 7 |  |
| Horn-Ross and colleagues, 2002 | Population-based case-control study, USA | 608/558 | F, 20-74 y | FFQ | Genistein  Daidzein  Biochanin A  Formononetin  Total isoflavones | Q5 (≥3,681 ug/d) vs. Q1 (<525 ug/d)  Q5 (≥3,597 ug/d) vs. Q1 (<469 ug/d)  Q5 (≥52 ug/d) vs. Q1 (<11 ug/d)  Q5 (≥52 ug/d) vs. Q1 (<9 ug/d)  Q5 (≥7,286 ug/d) vs. Q1 (<1,046 ug/d) | Age, race/ethnicity, daily caloric intake, goiter or thyroid nodules, radiation to the head or neck, family history of proliferative thyroid disease, age at menarche, use of OC, age at first full-term pregnancy/nulliparity, menopausal status and number of pregnancies in the last 5 years among premenopausal women. | 8 |  |
| Zamora-Ros and colleagues, 2020 | Prospective cohort study, 1992-2014 (mean 13.9 y), Europe | 748/476,108 | M&F,  35-70 y | FFQ | Flavonoids  Flavanols  Flavonols  Flavanones  Anthocyanins  Flavones  Isoflavonoids | Q4 (>689.6 mg/d) vs. Q1 (<254.4 mg/d)  Q4 (>516.9 mg/d) vs. Q1 (<157.8 mg/d)  Q4 (>53.2 mg/d) vs. Q1 (<16.1 mg/d)  Q4 (>55.4 mg/d) vs. Q1 (<10.3 mg/d)  Q4 (>51.7 mg/d) vs. Q1 (<12.4 mg/d)  Q4 (>14.8 mg/d) vs. Q1 (<5.8 mg/d)  Q4 (>0.1 mg/d) vs. Q1 (<0.0 mg/d) | Sex, age, center, smoking status, education level, BMI, physical activity, TEI and alcohol intakes, and menopausal status, OC use and infertility problems in women. | 8 |  |
| Abbreviations: 4-DFR, 4-day food recordings; AFFQ, Arizona food frequency questionnaire; BMI, body mass index; BMR, basal metabolic rate; CSDQ, country- or center-specific dietary questionnaires; DHBQ, dietary habits questionnaire; DHTQ, dietary history questionnaire; ER, estrogen receptor; F, female; FFQ, food frequency questionnaire; HRT, hormone replacement therapy; LOD, limit of detection; M, male; NR, not reported; OC, oral contraceptive; PHC, public health centre; PUFA, polyunsaturated fatty acid; QFFQ, quantitative food frequency questionnaire; SFFQ, semi-quantitative food frequency questionnaire; TEI, total energy intake. | | | | | | | | | |

^a^ The comparison between the highest category of exposure and the lowest (reference) were used in the meta-analysis, e.g. Q5 (the highest quintile) vs. Q1 (the lowest quintile), Q4 (the highest quartile) vs. Q1 (the lowest quartile), T3 (the highest tertile) vs. T1 (the lowest tertile).

^b^ NOS scores were computed by using the Newcastle-Ottawa Scale (NOS) quality assessment criteria for a total score of 9 points (9 representing the highest quality).

**Supplementary Table S****2**. Summary of Newcastle-Ottawa Scale (NOS) scores of cohort studies on flavonoid consumption and the risk of hormone-related cancers included in the meta-analysis.

| Author, year | Selection | | | | Comparability | Outcome | | | Total NOS scores^a^ |
| --- | --- | --- | --- | --- | --- | --- | --- | --- | --- |
|  | Representativeness of the exposed cohort | Selection of the unexposed cohort | Ascertainment of exposure | Outcome of interest not present at start of study | Comparability of cohorts | Assessment of outcome | Follow-up long enough for outcomes | Adequacy of follow-up of cohorts |  |
| *Breast* |  |  |  |  |  |  |  |  |  |
| Knekt and colleagues, 2002 | * | * | * | * | ** | * | * |  | 8 |
| Adebamowo and colleagues, 2005 | * | * | * | * | * | * | * |  | 7 |
| Touvier and colleagues, 2013 | * | * | * | * | ** | * | * | * | 9 |
| Zamora-Ros and colleagues, 2013 | * | * | * | * | ** | * | * |  | 8 |
| Wang and colleagues, 2014 | * | * | * | * | ** | * | * | * | 9 |
| Wang and colleagues, 2009 | * | * | * | * | ** | * | * |  | 8 |
| Kyrø and colleagues, 2015 | * | * | * | * | * | * | * |  | 7 |
| Pantavos and colleagues, 2015 | * | * | * | * | ** | * | * |  | 8 |
| Fink and colleagues, 2007 | * | * | * | * | * | * | * | * | 8 |
| *Endometrium* |  |  |  |  |  |  |  |  |  |
| Ollberding and colleagues, 2012 | * | * | * | * | ** | * | * |  | 8 |
| Budhathoki and colleagues, 2015 | * | * | * | * | ** | * | * | * | 9 |
| Wang and colleagues, 2009 | * | * | * | * | ** | * | * |  | 8 |
| *Ovary* |  |  |  |  |  |  |  |  |  |
| Chang and colleagues, 2007 |  | * | * | * | * | * | * |  | 6 |
| Gates and colleagues, 2007 |  | * | * | * | ** | * | * |  | 7 |
| Hedelin and colleagues, 2011 | * | * | * | * | ** | * | * |  | 8 |
| Cassidy and colleagues, 2014 | * | * | * | * | ** | * | * |  | 8 |
| Wang and colleagues, 2009 | * | * | * | * | ** | * | * |  | 8 |
| *Prostate* |  |  |  |  |  |  |  |  |  |
| Knekt and colleagues, 2002 | * | * | * | * | ** | * | * |  | 8 |
| Mursu and colleagues, 2008 | * | * | * | * | ** | * | * | * | 9 |
| Park and colleagues, 2008 | * | * | * | * | * | * | * | * | 8 |
| Geybels and colleagues, 2013 | * | * | * | * | * | * | * | * | 8 |
| Wang and colleagues, 2014 | * | * | * | * | ** | * | * | * | 9 |
| Reger and colleagues, 2018 | * | * | * | * | ** | * | * |  | 8 |
| *Thyroid* |  |  |  |  |  |  |  |  |  |
| Xiao and colleagues, 2014 | * | * | * | * | * | * | * |  | 7 |
| Zamora-Ros and colleagues, 2020 | * | * | * | * | ** | * | * |  | 8 |

^a^ Total NOS scores were computed by using the Newcastle-Ottawa Scale (NOS) quality assessment criteria for a total score of 9 points (9 representing the highest quality). A study can be awarded a maximum of one star for each numbered item within the Selection and Outcome categories. A maximum of two stars can be given for Comparability.

**Supplementary Table S3**. Summary of Newcastle-Ottawa Scale (NOS) scores of case-control and nested case-control studies on flavonoid consumption and the risk of hormone-related cancers included in the meta-analysis.

| Author, year | Selection | | | | | Comparability | | Exposure | | | Total NOS scores^a^ |
| --- | --- | --- | --- | --- | --- | --- | --- | --- | --- | --- | --- |
|  | Adequate definition of cases | Representativeness of the cases | Selection of controls | Definition of controls | Comparability of cases and controls | | Ascertainment of exposure | | Same method of ascertainment for all subjects | Non-response rate |  |
| *Breast* |  |  |  |  |  | |  | |  |  |  |
| Horn-Ross and colleagues, 2001 | * | * | * | * | ** | |  | | * | * | 8 |
| Linseisen and colleagues, 2004 | * | * | * |  | * | |  | | * |  | 5 |
| Bosetti and colleagues, 2005 | * | * |  | * | ** | |  | | * |  | 6 |
| Fink and colleagues, 2007 |  | * | * | * | ** | |  | | * |  | 6 |
| Torres-Sanchez and colleagues, 2008 |  | * |  | * | ** | |  | | * |  | 5 |
| Li and colleagues, 2013 | * |  | * | * | * | |  | | * |  | 5 |
| Luo and colleagues, 2010 | * | * | * | * | ** | | * | | * | * | 9 |
| Dai and colleagues, 2002 |  | * | * | * | * | | * | | * | * | 7 |
| Zheng and colleagues, 1999 |  | * | * | * | * | | * | | * | * | 7 |
| Feng and colleagues, 2019 | * | * |  | * | ** | |  | | * | * | 7 |
| Feng and colleagues, 2021 | * | * |  | * | ** | | * | | * | * | 8 |
| *Endometrium* |  |  |  |  |  | |  | |  |  |  |
| Horn-Ross and colleagues, 2013 |  | * | * | * | ** | |  | | * | * | 7 |
| Xu and colleagues, 2008 |  | * | * | * | ** | |  | | * |  | 6 |
| Bandera and colleagues, 2009 |  | * | * | * | ** | |  | | * | * | 7 |
| Rossi and colleagues, 2013 |  | * |  | * | ** | |  | | * | * | 6 |
| Neill and colleagues, 2014 | * | * | * | * | ** | |  | | * | * | 8 |
| *Ovary* |  |  |  |  |  | |  | |  |  |  |
| Rossi and colleagues, 2008 | * | * |  | * | * | | * | | * | * | 7 |
| Gates and colleagues, 2009 | * | * | * | * | * | | * | | * | * | 8 |
| Bandera and colleagues, 2011 | * | * | * | * | ** | |  | | * | * | 8 |
| Lee and colleagues, 2014 | * | * |  | * | ** | | * | | * | * | 8 |
| Neill and colleagues, 2014 | * | * | * | * | ** | |  | | * | * | 8 |
| Zhang and colleagues, 2004 | * |  |  | * | ** | |  | | * | * | 6 |
| *Prostate* |  |  |  |  |  | |  | |  |  |  |
| Bosetti and colleagues, 2006 | * |  |  | * | ** | |  | | * | * | 6 |
| Hedelin and colleagues, 2006 | * |  | * | * | ** | |  | | * |  | 6 |
| Heald and colleagues, 2007 | * |  | * | * | ** | | * | | * |  | 7 |
| Nagata and colleagues, 2007 | * | * |  | * | * | |  | | * |  | 5 |
| Reale and colleagues, 2018  Russo and colleagues, 2018 |  |  | * | * | ** | |  | | * |  | 5 |
| Ghanavati and colleagues, 2020 | * | * |  | * | ** | |  | | * | * | 7 |
| *Testis* |  |  |  |  |  | |  | |  |  |  |
| Walcott and colleagues, 2002 | * | * | * | * | ** | |  | | * | * | 8 |
| *Thyroid* |  |  |  |  |  | |  | |  |  |  |
| Horn-Ross and colleagues, 2002 | * | * | * | * | ** | |  | | * | * | 8 |

^a^ Total NOS scores were computed by using the Newcastle-Ottawa Scale (NOS) quality assessment criteria for a total score of 9 points (9 representing the highest quality). A study can be awarded a maximum of one star for each numbered item within the Selection and Exposure categories. A maximum of two stars can be given for Comparability.

**Supplementary Table S4**. Summary risk estimates and subgroup analyses based on study design for the association between flavonoid consumption and the risk of hormone-related cancers.

|  |  | Overall | | | | Prospective | | | | Case-control | | | |
| --- | --- | --- | --- | --- | --- | --- | --- | --- | --- | --- | --- | --- | --- |
|  | Total/Subclass of/Individual flavonoids | No. of Datasets (Studies) | OR^a^ (95% CI^b^) | *I^2^* (%)^c^ | *p*^c^ | No. of Datasets (Studies) | OR^a^ (95% CI^b^) | *I^2^* (%)^c^ | *p*^c^ | No. of Datasets (Studies) | OR^a^ (95% CI^b^) | *I^2^* (%)^c^ | *p*^c^ |
| *Breast Cancer* | **Total flavonoids** | 12 (10) | 0.94 (0.85-1.05) | 64.6 | 0.001 | 10 (8) | 0.99 (0.90-1.08) | 48.7 | 0.041 | 2 (2) | 0.70 (0.56-0.89) | 15.5 | 0.277 |
|  | **Flavonols** | 14 (12) | 0.85 (0.76-0.96) | 76.7 | <0.001 | 9 (7) | 0.97 (0.89-1.05) | 38.3 | 0.109 | 5 (5) | 0.66 (0.54-0.81) | 63.0 | 0.029 |
|  | Kaempferol | 4 (4) | 0.87 (0.65-1.16) | 68.7 | 0.022 |  |  |  |  |  |  |  |  |
|  | Myricetin | 2 (2) | 0.98 (0.79-1.22) | 0.0 | 0.887 |  |  |  |  |  |  |  |  |
|  | Quercetin | 4 (4) | 0.90 (0.74-1.10) | 33.8 | 0.210 |  |  |  |  |  |  |  |  |
|  | **Flavones** | 12 (10) | 0.85 (0.75-0.96) | 81.0 | <0.001 | 7 (5) | 0.96 (0.87-1.05) | 53.3 | 0.046 | 5 (5) | 0.71 (0.54-0.93) | 81.2 | <0.001 |
|  | **Flavanones** | 12 (10) | 0.96 (0.91-1.02) | 43.5 | 0.053 | 7 (5) | 1.00 (0.97-1.03) | 0.0 | 0.974 | 5 (5) | 0.82 (0.70-0.97) | 50.3 | 0.090 |
|  | Hesperetin | 3 (3) | 0.89 (0.71-1.12) | 0.0 | 0.743 |  |  |  |  |  |  |  |  |
|  | Naringenin | 3 (3) | 0.78 (0.41-1.50) | 86.4 | 0.001 |  |  |  |  |  |  |  |  |
|  | **Anthocyanidins** | 10 (8) | 0.94 (0.85-1.04) | 74.4 | <0.001 | 7 (5) | 0.99 (0.91-1.08) | 57.3 | 0.029 | 3 (3) | 0.84 (0.60-1.19) | 86.1 | 0.001 |
|  | **Flavan-3-ols** | 11 (10) | 0.97 (0.92-1.02) | 11.7 | 0.333 | 6 (5) | 0.98 (0.93-1.02) | 0.0 | 0.866 | 5 (5) | 0.95 (0.80-1.14) | 55.1 | 0.063 |
|  | Catechins | 3 (2) | 1.04 (0.53-2.06) | 79.3 | 0.008 |  |  |  |  |  |  |  |  |
|  | Epicatechin | 2 (2) | 1.11 (0.92-1.32) | 0.0 | 0.490 |  |  |  |  |  |  |  |  |
|  | Epigallocatechin | 2 (2) | 1.11 (0.75-1.64) | 71.9 | 0.059 |  |  |  |  |  |  |  |  |
|  | Theaflavins | 4 (3) | 1.02 (0.85-1.22) | 54.5 | 0.086 |  |  |  |  |  |  |  |  |
|  | Flavan-3-ol monomers | 2 (2) | 1.01 (0.94-1.09) | 0.0 | 0.930 |  |  |  |  |  |  |  |  |
|  | **Isoflavones** | 14 (13) | 0.96 (0.92-1.01) | 61.9 | 0.001 | 5 (4) | 1.00 (0.99-1.01) | 0.0 | 0.960 | 9 (9) | 0.79 (0.67-0.94) | 61.7 | 0.007 |
|  | Daidzein | 6 (6) | 0.65 (0.46-0.92) | 76.2 | 0.001 |  |  |  |  |  |  |  |  |
|  | Genistein | 6 (6) | 0.61 (0.44-0.83) | 68.0 | 0.008 |  |  |  |  |  |  |  |  |
|  | Glycitein | 5 (5) | 0.63 (0.44-0.91) | 58.6 | 0.047 |  |  |  |  |  |  |  |  |
|  | Biochanin A | 2 (2) | 1.07 (0.78-1.47) | 32.2 | 0.224 |  |  |  |  |  |  |  |  |
|  | Formononetin | 3 (3) | 0.91 (0.52-1.57) | 90.4 | <0.001 |  |  |  |  |  |  |  |  |
| *Ovarian Cancer* | **Total flavonoids** | 5 (5) | 0.95 (0.82-1.09) | 0.0 | 0.451 | 3 (3) | 0.84 (0.69-1.03) | 0.0 | 0.588 | 2 (2) | 1.07 (0.87-1.30) | 0.0 | 0.964 |
|  | **Flavonols** | 2 (2) | 0.70 (0.58-0.85) | 0.0 | 0.340 |  |  |  |  |  |  |  |  |
|  | Kaempferol | 2 (2) | 0.69 (0.49-0.96) | 0.0 | 0.345 |  |  |  |  |  |  |  |  |
|  | Myricetin | 2 (2) | 1.20 (0.86-1.68) | 0.0 | 0.367 |  |  |  |  |  |  |  |  |
|  | Quercetin | 2 (2) | 1.07 (0.77-1.47) | 0.0 | 0.910 |  |  |  |  |  |  |  |  |
|  | **Flavones** | 2 (2) | 0.83 (0.69-1.00) | 0.0 | 0.608 |  |  |  |  |  |  |  |  |
|  | Apigenin | 2 (2) | 1.04 (0.50-2.15) | 90.0 | 0.002 |  |  |  |  |  |  |  |  |
|  | Luteolin | 2 (2) | 0.75 (0.43-1.29) | 65.8 | 0.087 |  |  |  |  |  |  |  |  |
|  | **Flavanones** | 2 (2) | 1.00 (0.62-1.61) | 85.9 | 0.008 |  |  |  |  |  |  |  |  |
|  | **Anthocyanidins** | 2 (2) | 0.97 (0.81-1.16) | 0.0 | 0.821 |  |  |  |  |  |  |  |  |
|  | **Flavan-3-ols** | 2 (2) | 0.90 (0.75-1.08) | 0.0 | 0.907 |  |  |  |  |  |  |  |  |
|  | **Isoflavones** | 7 (7) | 0.67 (0.50-0.89) | 71.3 | 0.002 | 2 (2) | 0.85 (0.38-1.88) | 80.1 | 0.025 | 5 (5) | 0.61 (0.46-0.83) | 69.0 | 0.012 |
|  | Daidzein | 5 (5) | 0.66 (0.48-0.91) | 70.0 | 0.010 |  |  |  |  |  |  |  |  |
|  | Genistein | 5 (5) | 0.64 (0.46-0.88) | 69.5 | 0.011 |  |  |  |  |  |  |  |  |
|  | Glycitein | 4 (4) | 0.61 (0.41-0.91) | 74.2 | 0.009 |  |  |  |  |  |  |  |  |
|  | Formononetin | 2 (2) | 0.70 (0.54-0.89) | 0.0 | 0.888 |  |  |  |  |  |  |  |  |
| *Endometrial* | **Total flavonoids** | 2 (2) | 0.93 (0.67-1.27) | 32.7 | 0.223 |  |  |  |  |  |  |  |  |
| *Cancer* | **Isoflavones** | 7 (7) | 0.81 (0.70-0.94) | 22.7 | 0.256 | 2 (2) | 0.80 (0.51-1.25) | 53.7 | 0.142 | 5 (5) | 0.82 (0.69-0.97) | 24.6 | 0.258 |
|  | Daidzein | 4 (4) | 0.78 (0.60-1.03) | 51.2 | 0.074 |  |  |  |  |  |  |  |  |
|  | Genistein | 4 (4) | 0.81 (0.62-1.06) | 50.5 | 0.109 |  |  |  |  |  |  |  |  |
|  | Glycitein | 3 (3) | 0.84 (0.68-1.02) | 0.0 | 0.630 |  |  |  |  |  |  |  |  |
|  | Biochanin A | 2 (2) | 1.09 (0.86-1.40) | 0.0 | 0.973 |  |  |  |  |  |  |  |  |
|  | Formononetin | 3 (3) | 0.97 (0.78-1.22) | 0.0 | 0.844 |  |  |  |  |  |  |  |  |
| *Thyroid Cancer* | **Total flavonoids** | 2 (2) | 0.97 (0.75-1.24) | 42.8 | 0.186 |  |  |  |  |  |  |  |  |
|  | **Flavonols** | 2 (2) | 0.89 (0.73-1.09) | 17.3 | 0.272 |  |  |  |  |  |  |  |  |
|  | **Flavones** | 2 (2) | 1.24 (1.03-1.50) | 0.0 | 0.835 |  |  |  |  |  |  |  |  |
|  | **Flavanones** | 2 (2) | 1.31 (1.09-1.57) | 9.5 | 0.293 |  |  |  |  |  |  |  |  |
|  | **Anthocyanidins** | 2 (2) | 1.12 (0.93-1.36) | 0.0 | 0.745 |  |  |  |  |  |  |  |  |
|  | **Flavan-3-ols** | 2 (2) | 0.81 (0.54-1.22) | 78.4 | 0.032 |  |  |  |  |  |  |  |  |
|  | **Isoflavones** | 3 (3) | 0.90 (0.74-1.11) | 25.0 | 0.263 |  |  |  |  |  |  |  |  |
| *Prostate Cancer* | **Total flavonoids** | 6 (6) | 1.11 (1.02-1.21) | 0.0 | 0.484 | 3 (3) | 1.11 (1.01-1.22) | 0.0 | 0.994 | 3 (3) | 0.90 (0.48-1.70) | 55.2 | 0.108 |
|  | **Flavonols** | 6 (6) | 1.02 (0.85-1.23) | 58.7 | 0.033 | 3 (3) | 1.07 (0.98-1.17) | 0.0 | 0.607 | 3 (3) | 0.61 (0.22-1.70) | 82.0 | 0.004 |
|  | Kaempferol | 2 (2) | 0.98 (0.84-1.16) | 0.0 | 0.888 |  |  |  |  |  |  |  |  |
|  | Myricetin | 2 (2) | 0.67 (0.33-1.34) | 75.7 | 0.042 |  |  |  |  |  |  |  |  |
|  | Quercetin | 2 (2) | 0.99 (0.84-1.17) | 0.0 | 0.395 |  |  |  |  |  |  |  |  |
|  | **Flavones** | 5 (5) | 0.96 (0.77-1.18) | 45.1 | 0.121 | 2 (2) | 0.96 (0.70-1.31) | 46.8 | 0.170 | 3 (3) | 0.77 (0.39-1.50) | 62.6 | 0.069 |
|  | **Flavanones** | 5 (5) | 1.01 (0.65-1.56) | 85.1 | <0.001 | 2 (2) | 1.08 (0.98-1.89) | 0.0 | 0.948 | 3 (3) | 0.85 (0.16-4.55) | 92.3 | <0.001 |
|  | **Anthocyanidins** | 5 (5) | 1.07 (0.86-1.34) | 46.6 | 0.112 | 2 (2) | 1.11 (1.01-1.22) | 0.0 | 0.344 | 3 (3) | 0.70 (0.32-1.52) | 69.0 | 0.040 |
|  | **Flavan-3-ols** | 5 (5) | 1.11 (0.86-1.45) | 57.2 | 0.053 | 2 (2) | 1.18 (1.07-1.30) | 0.0 | 0.862 | 3 (3) | 0.76 (0.21-2.79) | 78.5 | 0.010 |
|  | Catechins | 2 (2) | 0.37 (0.05-2.83) | 92.6 | <0.001 |  |  |  |  |  |  |  |  |
|  | **Isoflavones** | 8 (8) | 1.02 (0.93-1.12) | 42.8 | 0.093 | 3 (3) | 1.04 (0.93-1.16) | 67.3 | 0.047 | 5 (5) | 0.96 (0.79-1.17) | 25.5 | 0.251 |
|  | Daidzein | 6 (6) | 1.02 (0.88-1.18) | 49.1 | 0.080 |  |  |  |  |  |  |  |  |
|  | Genistein | 6 (6) | 0.96 (0.89-1.03) | 0.0 | 0.512 |  |  |  |  |  |  |  |  |
|  | Glycitein | 3 (3) | 0.97 (0.81-1.16) | 66.1 | 0.052 |  |  |  |  |  |  |  |  |
|  | Biochanin A | 2 (2) | 1.04 (0.92-1.17) | 0.0 | 0.465 |  |  |  |  |  |  |  |  |
| *Women-specific* | **Total flavonoids** | 19 (15) | 0.94 (0.87-1.02) | 50.8 | 0.006 | 14 (10) | 0.97 (0.90-1.05) | 39.0 | 0.067 | 5 (5) | 0.87 (0.71-1.08) | 61.9 | 0.033 |
| *Cancers* | **Flavonols** | 17 (15) | 0.85 (0.76-0.94) | 75.5 | <0.001 | 10 (8) | 0.95 (0.88-1.03) | 44.2 | 0.064 | 7 (7) | 0.71 (0.58-0.86) | 72.6 | 0.001 |
|  | Kaempferol | 6 (6) | 0.81 (0.65-1.03) | 58.4 | 0.034 |  |  |  |  |  |  |  |  |
|  | Myricetin | 4 (4) | 1.04 (0.87-1.25) | 0.0 | 0.607 |  |  |  |  |  |  |  |  |
|  | Quercetin | 7 (7) | 0.90 (0.77-1.06) | 21.5 | 0.265 |  |  |  |  |  |  |  |  |
|  | **Flavones** | 15 (13) | 0.85 (0.77-0.95) | 76.3 | <0.001 | 8 (6) | 0.95 (0.87-1.03) | 48.0 | 0.062 | 7 (7) | 0.75 (0.61-0.91) | 74.6 | 0.001 |
|  | **Flavanones** | 15 (13) | 0.95 (0.89-1.01) | 51.5 | 0.011 | 8 (6) | 0.99 (0.96-1.03) | 0.0 | 0.648 | 7 (7) | 0.87 (0.74-1.03) | 65.1 | 0.009 |
|  | **Anthocyanidins** | 13 (11) | 0.96 (0.88-1.04) | 66.4 | <0.001 | 8 (6) | 0.99 (0.92-1.07) | 50.8 | 0.048 | 5 (5) | 0.91 (0.73-1.15) | 77.6 | 0.001 |
|  | **Flavan-3-ols** | 14 (13) | 0.97 (0.93-1.01) | 0.0 | 0.532 | 7 (6) | 0.97 (0.93-1.02) | 0.0 | 0.902 | 7 (7) | 0.94 (0.83-1.07) | 34.7 | 0.163 |
|  | **Isoflavones** | 28 (26) | 0.87 (0.82-0.92) | 73.8 | <0.001 | 9 (8) | 1.00 (0.97-1.03) | 34.5 | 0.142 | 19 (18) | 0.75 (0.66-0.85) | 62.9 | <0.001 |
|  | Daidzein | 15 (14) | 0.69 (0.58-0.83) | 67.1 | <0.001 |  |  |  |  |  |  |  |  |
|  | Genistein | 15 (14) | 0.67 (0.57-0.80) | 63.5 | <0.001 |  |  |  |  |  |  |  |  |
|  | Glycitein | 12 (11) | 0.68 (0.57-0.83) | 58.5 | 0.005 |  |  |  |  |  |  |  |  |
|  | Biochanin A | 5 (4) | 1.02 (0.88-1.18) | 0.0 | 0.440 |  |  |  |  |  |  |  |  |
|  | Formononetin | 8 (7) | 0.87 (0.69-1.10) | 72.4 | 0.001 |  |  |  |  |  |  |  |  |
| *Men-specific* | **Total flavonoids** | 7 (7) | 1.11 (1.02-1.22) | 0.0 | 0.537 | 3 (3) | 1.11 (1.01-1.22) | 0.0 | 0.994 | 4 (4) | 1.01 (0.59-1.71) | 40.3 | 0.170 |
| *Cancers* | **Isoflavones** | 9 (9) | 1.02 (0.93-1.11) | 35.8 | 0.132 | 3 (3) | 1.04 (0.93-1.16) | 67.3 | 0.047 | 6 (6) | 0.96 (0.81-1.14) | 8.8 | 0.360 |

^a^ OR=odds ratio.

^b^ CI=confidence interval.

^c^ Statistic *I^2^* was used for the assessment of heterogeneity across the studies and the *p* values (two-sided) based on the *Q* test of heterogeneity were reported (*p* < 0.05 suggested significant heterogeneity).

**Supplementary Table S5**. Subgroup analyses based on menopausal status for the association between flavonoid consumption and breast cancer risk.

|  |  | Premenopausal | | | | Postmenopausal | | | |
| --- | --- | --- | --- | --- | --- | --- | --- | --- | --- |
|  | Total/Subclass of flavonoids | No. of Datasets (Studies) | OR^a^ (95% CI^b^) | *I^2^* (%)^c^ | *p*^c^ | No. of Datasets (Studies) | OR^a^ (95% CI^b^) | *I^2^* (%)^c^ | *p*^c^ |
| *Breast Cancer* | Total flavonoids | 4 (4) | 1.02 (0.90-1.15) | 0.0 | 0.498 | 5 (5) | 0.95 (0.87-1.05) | 39.9 | 0.155 |
|  | Flavonols | 8 (8) | 0.95 (0.84-1.08) | 49.5 | 0.054 | 8 (8) | 0.88 (0.78-0.98) | 72.0 | 0.001 |
|  | Flavones | 7 (7) | 0.89 (0.82-0.97) | 0.0 | 0.526 | 8 (8) | 0.91 (0.81-1.01) | 69.4 | 0.002 |
|  | Flavanones | 7 (7) | 0.97 (0.88-1.07) | 32.7 | 0.178 | 8 (8) | 0.99 (0.96-1.03) | 0.0 | 0.664 |
|  | Anthocyanidins | 4 (4) | 1.28 (1.00-1.18) | 0.0 | 0.725 | 5 (5) | 0.97 (0.91-1.04) | 23.2 | 0.266 |
|  | Flavan-3-ols | 7 (7) | 1.04 (0.93-1.17) | 39.7 | 0.127 | 8 (8) | 0.94 (0.89-1.00) | 22.8 | 0.248 |
|  | Isoflavones | 9 (9) | 0.98 (0.91-1.05) | 17.6 | 0.286 | 9 (9) | 1.00 (0.98-1.01) | 0.0 | 0.690 |

^a^ OR=odds ratio.

^b^ CI=confidence interval.

^c^ Statistic *I^2^* was used for the assessment of heterogeneity across the studies and the *p* values (two-sided) based on the *Q* test of heterogeneity were reported (*p* < 0.05 suggested significant heterogeneity).

**Supplementary Table S6**. Subgroup analyses based on study region for the association between flavonoid consumption and the risk of hormone-related cancers.

|  |  | Non-Asia | | | | Asia | | | |
| --- | --- | --- | --- | --- | --- | --- | --- | --- | --- |
|  | Total/Subclass of flavonoids | No. of Datasets (Studies) | OR^a^ (95% CI^b^) | *I^2^* (%)^c^ | *p*^c^ | No. of Datasets (Studies) | OR^a^ (95% CI^b^) | *I^2^* (%)^c^ | *p*^c^ |
| *Breast Cancer* | Total flavonoids | 11 (9) | 0.98 (0.91-1.07) | 43.7 | 0.059 | 1 (1) | 0.66 (0.54-0.82) | NA^d^ | NA |
|  | Flavonols | 11 (9) | 0.92 (0.84-1.01) | 54.3 | 0.016 | 3 (3) | 0.69 (0.47-1.01) | 81.7 | 0.004 |
|  | Flavones | 10 (8) | 0.91 (0.83-1.00) | 57.6 | 0.012 | 2 (2) | 0.67 (0.35-1.30) | 94.8 | <0.001 |
|  | Flavanones | 9 (7) | 0.99 (0.96-1.03) | 0.0 | 0.967 | 3 (3) | 0.74 (0.60-0.91) | 34.2 | 0.219 |
|  | Anthocyanidins | 9 (7) | 0.99 (0.93-1.07) | 47.6 | 0.054 | 1 (1) | 0.61 (0.49-0.75) | NA | NA |
|  | Flavan-3-ols | 8 (7) | 0.96 (0.92-1.01) | 0.0 | 0.733 | 3 (3) | 1.10 (0.83-1.46) | 64.7 | 0.059 |
|  | Isoflavones | 9 (8) | 1.00 (0.99-1.01) | 0.0 | 0.992 | 5 (5) | 0.65 (0.56-0.76) | 0.0 | 0.536 |
| *Ovarian Cancer* | Total flavonoids | 5 (5) | 0.95 (0.82-1.09) | 0.0 | 0.451 | NA | NA | NA | NA |
|  | Isoflavones | 5 (5) | 0.76 (0.55-1.05) | 69.8 | 0.010 | 2 (2) | 0.47 (0.35-0.63) | 0.0 | 0.691 |
| *Endometrial Cancer* | Isoflavones | 5 (5) | 0.80 (0.65-0.98) | 38.5 | 0.164 | 2 (2) | 0.82 (0.62-1.07) | 19.7 | 0.264 |
| *Prostate Cancer* | Total flavonoids | 5 (5) | 1.12 (1.02-1.23) | 0.0 | 0.991 | 1 (1) | 0.39 (0.14-1.05) | NA | NA |
|  | Flavonols | 5 (5) | 1.03 (0.86-1.25) | 64.5 | 0.024 | 1 (1) | 0.70 (0.28-1.73) | NA | NA |
|  | Flavones | 4 (4) | 1.04 (0.95-1.14) | 0.0 | 0.540 | 1 (1) | 0.33 (0.12-0.87) | NA | NA |
|  | Flavanones | 4 (4) | 1.18 (0.87-1.61) | 72.9 | 0.011 | 1 (1) | 0.10 (0.03-0.31) | NA | NA |
|  | Anthocyanidins | 4 (4) | 1.12 (0.92-1.37) | 42.5 | 0.157 | 1 (1) | 0.54 (0.21-1.35) | NA | NA |
|  | Flavan-3-ols | 4 (4) | 1.19 (1.09-1.31) | 0.0 | 0.885 | 1 (1) | 0.24 (0.08-0.67) | NA | NA |
|  | Isoflavones | 7 (7) | 1.04 (0.97-1.11) | 13.5 | 0.327 | 1 (1) | 0.48 (0.25-0.93) | NA | NA |
| *Women-specific* | Total flavonoids | 18 (14) | 0.97 (0.91-1.04) | 27.9 | 0.132 | 1 (1) | 0.66 (0.54-0.82) | NA | NA |
| *Cancers* | Flavonols | 14 (12) | 0.90 (0.82-0.98) | 60.2 | 0.002 | 3 (3) | 0.69 (0.47-1.01) | 81.7 | 0.004 |
|  | Flavones | 13 (11) | 0.90 (0.84-0.97) | 48.4 | 0.026 | 2 (2) | 0.67 (0.35-1.30) | 94.8 | <0.001 |
|  | Flavanones | 12 (10) | 0.99 (0.95-1.03) | 8.9 | 0.359 | 3 (3) | 0.74 (0.60-0.91) | 34.2 | 0.219 |
|  | Anthocyanidins | 12 (10) | 1.00 (0.94-1.05) | 30.3 | 0.149 | 1 (1) | 0.61 (0.49-0.75) | NA | NA |
|  | Flavan-3-ols | 11 (10) | 0.96 (0.92-1.00) | 0.0 | 0.896 | 3 (3) | 1.10 (0.83-1.46) | 64.7 | 0.059 |
|  | Isoflavones | 19 (17) | 0.96 (0.92-1.01) | 54.4 | 0.002 | 9 (9) | 0.64 (0.55-0.75) | 38.8 | 0.109 |
| *Men-specific* | Total flavonoids | 6 (6) | 1.12 (1.03-1.23) | 0.0 | 0.974 | 1 (1) | 0.39 (0.14-1.05) | NA | NA |
| *Cancers* | Isoflavones | 8 (8) | 1.04 (0.98-1.10) | 2.3 | 0.412 | 1 (1) | 0.48 (0.25-0.93) | NA | NA |

^a^ OR=odds ratio.

^b^ CI=confidence interval.

^c^ Statistic *I^2^* was used for the assessment of heterogeneity across the studies and the *p* values (two-sided) based on the *Q* test of heterogeneity were reported (*p* < 0.05 suggested significant heterogeneity).

^d^ NA=not applicable.

**Supplementary Table S7**. Begg's and Egger’s test detecting publication bias for included studies on flavonoid consumption and the risk of hormone-related cancers.

|  | Total/Subclass of flavonoids | No. of Datasets  (Studies) | Begg's test | | Egger’s test | |
| --- | --- | --- | --- | --- | --- | --- |
|  |  |  | *Z*^a^ | *p_Z_*^a^ | *t* | *p_t_* |
| *Breast Cancer* | Total flavonoids | 12 (10) | 0.34 | 0.732 | -0.42 | 0.684 |
|  | Flavonols | 14 (12) | 0.99 | 0.324 | -1.20 | 0.252 |
|  | Flavones | 12 (10) | 1.03 | 0.304 | -1.21 | 0.253 |
|  | Flavanones | 12 (10) | 0.62 | 0.537 | -1.19 | 0.260 |
|  | Anthocyanidins | 10 (8) | 0.54 | 0.592 | -0.71 | 0.501 |
|  | Flavan-3-ols | 11 (10) | 0.16 | 0.876 | 0.18 | 0.862 |
|  | Isoflavones | 14 (13) | 2.85 | 0.004 | -2.90 | 0.013 |
| *Ovarian Cancer* | Total flavonoids | 5 (5) | 0.24 | 0.806 | 0.09 | 0.936 |
|  | Isoflavones | 7 (7) | 0.00 | 1.000 | 0.01 | 0.989 |
| *Endometrial Cancer* | Isoflavones | 7 (7) | 0.00 | 1.000 | 0.01 | 0.996 |
| *Prostate Cancer* | Total flavonoids | 6 (6) | 1.13 | 0.260 | -0.85 | 0.443 |
|  | Flavonols | 6 (6) | 1.50 | 0.133 | -1.74 | 0.156 |
|  | Flavones | 5 (5) | 1.71 | 0.086 | -2.05 | 0.132 |
|  | Flavanones | 5 (5) | 0.24 | 0.806 | -0.24 | 0.829 |
|  | Anthocyanidins | 5 (5) | 0.73 | 0.462 | -0.99 | 0.397 |
|  | Flavan-3-ols | 5 (5) | 0.24 | 0.806 | -0.79 | 0.488 |
|  | Isoflavones | 8 (8) | 0.62 | 0.536 | -1.10 | 0.314 |
| *Women-specific Cancers* | Total flavonoids | 19 (15) | 0.00 | 1.000 | -0.47 | 0.647 |
|  | Flavonols | 17 (15) | 1.19 | 0.232 | -1.40 | 0.182 |
|  | Flavones | 15 (13) | 0.69 | 0.488 | -1.44 | 0.173 |
|  | Flavanones | 15 (13) | 0.30 | 0.767 | -1.23 | 0.239 |
|  | Anthocyanidins | 13 (11) | 0.43 | 0.669 | -0.62 | 0.545 |
|  | Flavan-3-ols | 14 (13) | 0.11 | 0.913 | -0.06 | 0.956 |
|  | Isoflavones | 28 (26) | 1.92 | 0.055 | -4.70 | <0.001 |
| *Men-specific Cancers* | Total flavonoids | 7 (7) | 0.00 | 1.000 | -0.39 | 0.715 |
|  | Isoflavones | 9 (9) | 0.94 | 0.348 | -1.22 | 0.261 |

^a^ Both *Z* and *p_Z_* values were continually corrected.

**Supplementary Table S8**. Sensitivity analyses for the consumption of total flavonoids in hormone-related cancers by sequential removal of each study.

|  | Study Omitted^a^ | OR^b^ | 95% LCI^c^ | 95% UCI^d^ |
| --- | --- | --- | --- | --- |
| *Breast Cancer* | Knekt and colleagues, 2002 | 0.94 | 0.84 | 1.04 |
|  | Fink and colleagues (2), 2007 | 0.95 | 0.85 | 1.05 |
|  | Fink and colleagues (1), 2007 | 0.95 | 0.85 | 1.06 |
|  | Wang and colleagues, 2009 | 0.93 | 0.83 | 1.05 |
|  | Touvier and colleagues (a), 2013 | 0.96 | 0.87 | 1.05 |
|  | Touvier and colleagues (b), 2013 | 0.93 | 0.85 | 1.02 |
|  | Zomora-Ros and colleagues, 2013 | 0.94 | 0.82 | 1.07 |
|  | Wang and colleagues, 2014 | 0.94 | 0.84 | 1.06 |
|  | Pantavo and colleagues s, 2015 | 0.95 | 0.85 | 1.05 |
|  | Kyrø and colleagues (a), 2015 | 0.94 | 0.84 | 1.05 |
|  | Kyrø and colleagues (b), 2015 | 0.93 | 0.82 | 1.05 |
|  | Feng and colleagues, 2019 | 0.98 | 0.91 | 1.07 |
|  | Combined | 0.94 | 0.85 | 1.05 |
| *Ovarian Cancer* | Gates and colleagues, 2007 | 0.98 | 0.84 | 1.14 |
|  | Rossi and colleagues, 2008 | 0.90 | 0.76 | 1.06 |
|  | Gates and colleagues, 2009 | 0.92 | 0.78 | 1.08 |
|  | Wang and colleagues, 2009 | 0.94 | 0.80 | 1.10 |
|  | Cassidy and colleagues, 2014 | 0.99 | 0.84 | 1.18 |
|  | Combined | 0.95 | 0.82 | 1.09 |
| *Prostate Cancer* | Knekt and colleagues, 2002 | 1.11 | 0.97 | 1.26 |
|  | Bosetti and colleagues, 2006 | 1.11 | 0.97 | 1.24 |
|  | Mursu and colleagues, 2008 | 1.11 | 0.97 | 1.26 |
|  | Wang and colleagues, 2014 | 1.10 | 0.86 | 1.40 |
|  | Reale and colleagues, 2018 | 1.11 | 0.98 | 1.26 |
|  | Ghanavati and colleagues, 2020 | 1.12 | 1.02 | 1.23 |
|  | Combined | 1.11 | 1.02 | 1.21 |
| *Women-specific Cancers* | Knekt and colleagues, 2002 | 0.94 | 0.87 | 1.02 |
|  | Fink and colleagues (2), 2007 | 0.95 | 0.87 | 1.03 |
|  | Fink and colleagues (1), 2007 | 0.95 | 0.87 | 1.03 |
|  | Wang and colleagues, 2009 | 0.94 | 0.86 | 1.02 |
|  | Touvier and colleagues (a), 2013 | 0.95 | 0.89 | 1.03 |
|  | Touvier and colleagues (b), 2013 | 0.94 | 0.87 | 1.01 |
|  | Zamora-Ros and colleagues, 2013 | 0.94 | 0.85 | 1.03 |
|  | Wang and colleagues, 2014 | 0.94 | 0.86 | 1.03 |
|  | Pantavos and colleagues, 2015 | 0.94 | 0.87 | 1.03 |
|  | Kyrø and colleagues (a), 2015 | 0.94 | 0.86 | 1.02 |
|  | Kyrø and colleagues (b), 2015 | 0.93 | 0.85 | 1.02 |
|  | Feng and colleagues, 2019 | 0.97 | 0.91 | 1.04 |
|  | Wang and colleagues, 2009 | 0.94 | 0.87 | 1.02 |
|  | Rossi and colleagues, 2013 | 0.95 | 0.88 | 1.03 |
|  | Gates and colleagues, 2007 | 0.95 | 0.88 | 1.03 |
|  | Rossi and colleagues, 2008 | 0.94 | 0.86 | 1.02 |
|  | Gates and colleagues, 2009 | 0.94 | 0.86 | 1.02 |
|  | Wang and colleagues, 2009 | 0.94 | 0.87 | 1.02 |
|  | Cassidy and colleagues, 2014 | 0.95 | 0.87 | 1.03 |
|  | Combined | 0.94 | 0.87 | 1.02 |
| *Men-specific Cancers* | Knekt and colleagues, 2002 | 1.11 | 1.01 | 1.23 |
|  | Bosetti and colleagues, 2006 | 1.10 | 1.00 | 1.21 |
|  | Mursu and colleagues, 2008 | 1.11 | 1.01 | 1.22 |
|  | Wang and colleagues, 2014 | 1.13 | 0.91 | 1.39 |
|  | Reale and colleagues, 2018 | 1.11 | 1.01 | 1.22 |
|  | Ghanavati and colleagues, 2020 | 1.12 | 1.03 | 1.23 |
|  | Walcott and colleagues, 2002 | 1.11 | 1.02 | 1.21 |
|  | Combined | 1.11 | 1.02 | 1.22 |

^a^ Use (1) and (2) to distinguish two different studies of the same author in the same year; two datasets of the same study were represented by (a) and (b).

^b^ OR=odds ratio.

^c^ LCI=low confidence interval.

^d^ UCI=upper confidence interval.
